# Development and characterization of a new sunflower source of resistance to race G of *Orobanche cumana* Wallr. derived from *Helianthus anomalus*

**DOI:** 10.1007/s00122-024-04558-4

**Published:** 2024-02-22

**Authors:** Belén Fernández-Melero, Lidia del Moral, Marco Todesco, Loren H. Rieseberg, Gregory L. Owens, Sébastien Carrère, Mireille Chabaud, Stéphane Muños, Leonardo Velasco, Begoña Pérez-Vich

**Affiliations:** 1 Instituto de Agricultura Sostenible (IAS-CSIC), Alameda del Obispo S/N, 14004 Córdoba, Spain; 2 Department of Botany, University of British Columbia, Vancouver, BC V6T 1Z4 Canada; 3 Biodiversity Research Centre, University of British Columbia, Vancouver, BC V6T 1Z4 Canada; 4 Department of Biology, University of Victoria, Victoria, BC V8W 2Y2 Canada; 5 Laboratoire des Interactions Plantes Microbes-Environnement (LIPME), Université de Toulouse, CNRS, INRAE, Castanet-Tolosan, France

## Abstract

**Key message:**

**A new** *Or*_*Anom1*_** gene introgressed in cultivated sunflower from wild** ***Helianthus anomalus*** **confers late post-attachment resistance to** ***Orobanche cumana* race G and maps to a target interval in Chromosome 4 where two receptor-like kinases (RLKs) have been identified in the** ***H. anomalus*** **genome as putative candidates.**

**Abstract:**

Sunflower broomrape is a parasitic weed that infects sunflower (*Helianthus annuus* L.) roots causing severe yield losses. Breeding for resistance is the most effective and sustainable control method. In this study, we report the identification, introgression, and genetic and physiological characterization of a new sunflower source of resistance to race G of broomrape developed from the wild annual sunflower *H. anomalus* (accession PI 468642). Crosses between PI 468642 and the susceptible line P21 were carried out, and the genetic study was conducted in BC_1_F_1_, BC_1_F_2,_ and its derived BC_1_F_3_ populations. A BC_1_F_5_ germplasm named ANOM1 was developed through selection for race G resistance and resemblance to cultivated sunflower. The resistant trait showed monogenic and dominant inheritance. The gene, named *Or*_*Anom1*_, was mapped to Chromosome 4 within a 1.2 cM interval and co-segregated with 7 SNP markers. This interval corresponds to a 1.32 Mb region in the sunflower reference genome, housing a cluster of receptor-like kinase and receptor-like protein (RLK-RLP) genes. Notably, the analysis of the *H. anomalus* genome revealed the absence of RLPs in the *Or*_*Anom1*_ target region but featured two RLKs as possible *Or*_*Anom1*_ candidates. Rhizotron and histological studies showed that *Or*_*Anom1*_ determines a late post-attachment resistance mechanism. Broomrape can establish a vascular connection with the host, but parasite growth is stopped before tubercle development, showing phenolic compounds accumulation and tubercle necrosis. ANOM1 will contribute to broadening the genetic basis of broomrape resistance in the cultivated sunflower pool and to a better understanding of the molecular basis of the sunflower-broomrape interaction.

**Supplementary Information:**

The online version contains supplementary material available at 10.1007/s00122-024-04558-4.

## Introduction

Sunflower resistance to the parasitic weed *Orobanche cumana* Wallr., commonly known as sunflower broomrape, is mainly controlled by single genes showing major and dominant effects (Fernández-Martínez et al. 2015). The parasite virulence is also determined by dominant avirulence alleles at single loci (Rodríguez-Ojeda et al. 2013), following a gene-for-gene interaction scheme that leads to the emergence of different physiological races of the parasite, which can evolve in response to the host’s resistance mechanisms (Imerovski et al. 2016).

Although some studies have already been carried out, knowledge of the genomic location and physiological function of sunflower major genes conferring resistance to broomrape is still limited. Duriez et al. (2019) identified the *HaOr7* gene conferring resistance to sunflower race F, mapping it to Chromosome (Chr) 7 and showing that it encodes a leucine-rich repeat (LRR) receptor-like kinase. Fernández-Aparicio et al. (2022) located the *Or*_*Deb2*_ gene, which confers resistance to broomrape race G, in a genomic interval of Chr 4 containing a cluster of genes encoding LRR-receptor like proteins (RLPs) lacking a cytoplasmic kinase domain, and receptor-like kinases (RLKs) with one or two kinase domains and lacking an extracellular LRR region. Additionally, Martin-Sanz et al. (2020) mapped the *Or*_*SII*_ gene that provides late post-attachment resistance to broomrape races F and G to the same region of Chr 4. However, no comparative analysis of these resistance sources has been performed yet.

Other studies have identified the position of additional resistance genes, such as *Or5* gene conferring resistance to broomrape race E in the upper region of Chr 3 (Tang et al. 2003; Pérez-Vich et al. 2004). This region was also found to be a hotspot for resistance to more virulent populations (races F and G) in studies using QTL detection (Imerovski et al. 2019) and GWAS (Calderón-González et al. 2023). Furthermore, Imerovski et al. (2019) and Calderón-González et al. (2023) identified a second genomic region on Chr 3 associated with broomrape resistance, and Calderón-González et al. (2023) found potential genetic associations with broomrape resistance on Chrs 5, 10, 13, and 16. The biological function of these QTL and/or minor genes remains to be elucidated, although recent studies pointed the possible role in modulating sunflower *O. cumana* resistance responses of SWEET (Sugars Will Eventually be Exported Transporters) sugar transporter genes (Calderón-González et al. 2023), transcription factors of the basic leucine zipper family (Li et al. 2023) or genes involved in the biosynthesis of monophenols (*4CL2* 4-coumarate-CoA ligase (2) or basal defence regulation and salicylic acid signalling activation (EDS1, enhanced disease susceptibility 1) (Huang et al. 2022).

Sunflower broomrape virulence evolves rapidly. This parasitic weed has a high capacity of dispersion and mutation. Individual broomrape plants produce thousands of tiny seeds that are easily dispersed by wind and other agents, including sunflower seeds, to which broomrape seeds can be found attached (Fernández-Martínez et al. 2015). Vertical resistance mechanisms based on major genes described above have been repeatedly surpassed by the parasite (Fernández-Martínez et al. 2015). Consequently, breeders are constantly searching for new sources of resistance. Wild *Helianthus* species are a major reservoir of genes controlling resistance to the new virulent races of *O. cumana* (Christov et al. 2009; Seiler and Jan 2014; Chabaud et al. 2022). To ensure suitable sunflower production in areas affected by broomrape, identification and characterization of new genes conferring resistance to this parasitic weed are needed. The objectives of the present study were to (i) identify germplasm accessions of the wild annual sunflower species *H. anomalus* with resistance to broomrape race G and introgress the resistance into cultivated sunflower, (ii) carry out the genetic analysis of the resistance, including allelic crosses with the previously developed DEB2 line (Velasco et al. 2012), which also exhibits resistance to sunflower broomrape race G, (iii) develop a linkage map including the putative *Or* resistance gene using publicly SNP markers and new SNPs developed in this study, (iv) identify candidate genes underlying the resistance gene based on the *H. anomalus* chromosome level genome sequence, and (v) characterize the resistance mechanisms in the new developed material.

## Materials and methods

### Evaluation of *H. anomalus* accessions for resistance to sunflower broomrape and introgression of resistance into cultivated sunflower

Two accessions of *H. anomalus* S.F. Blake were provided in 2001 by the National Plant Germplasm System (NGPS) of the United States Department of Agriculture (USDA). The accessions were identified as PI 468638 (ANO-1495) and PI 468642 (ANO-1506), respectively. They were initially used for tocopherol analysis of the seeds (Velasco et al. 2003) and in 2014 for resistance to sunflower broomrape race G. The race G broomrape population (named GT) was collected in Çeşmekolu, Kirklareli Province, Turkey, in 2000. We used the Turkish population because no sunflower broomrape race G existed at the beginning of the research in the Guadalquivir Valley area, where the research was conducted. Shortly after, we identified race G populations in the area (Martín-Sanz et al. 2016), but since the study had been started with the Turkish population, the new Spanish race G populations had not been characterized yet in detail, and the amount of seed available was still very low, we decided to complete the study with the race G population from Turkey.

Seeds of the *H. anomalus* accessions were treated with gibberellic acid 1 mM for 1 h, then rinsed three times with water and maintained in a Petri dish with moistened filter paper in the dark at 25 ºC until they germinated. The plantlets were planted in small pots 7 × 7 × 7 cm filled with sand and peat and 30 mg of race G broomrape seeds. They were kept in a growth chamber at 25 ºC / 20 ºC (day/night) with 16 h photoperiod for eight weeks. After this time, they were transplanted into 6 L pots containing a soil mixture of sand, silt, and peat in a proportion 2:1:1 by volume and 8 g of NPK controlled release fertilizer Nutricote® 15–9-10 (2MgO) + ME. The pots were maintained in a greenhouse without control of temperature. Three plants of accession PI 468638 and four plants of accession PI 468642 reached flowering. None of them showed emerged broomrape shoots. Pollen from plants of each accession was used to pollinate male-sterile flowers of the nuclear male-sterile line P21 (Pérez-Vich et al. 2005), which is susceptible to race E (Pérez-Vich et al. 2004), race F (Pérez-Vich et al. 2002, 2004; Velasco et al. 2007; Akhtouch et al. 2008), and race G (this study) of *O. cumana*. *H. anomalus* plants were very weak and produced few small flowers. They were all self-incompatible, i.e. did not produce seeds under self-pollination conditions.

F_1_ seeds were sown following the same procedures described above, resulting in eight F_1_ plants for each of the accessions. All the F_1_ plants from the cross with PI 468638 showed emerged broomrape plants, whereas three out of the eight F_1_ plants from the cross with accession PI 468642 showed an absence of broomrape emergence at the flowering time. They were used to pollinate male-sterile plants of P21. The resistant F_1_ plants were bagged for self-fertilization, but they produced no seeds. Accordingly, BC_1_F_1_ seeds from a single backcross were sown in 2017 as described above, resulting in 12 resistant plants showing no broomrape emergence and 12 susceptible plants showing between 3 and 16 emerged broomrape shoots per plant. Resistant plants were self-pollinated and used for an additional backcross with P21. However, some of the BC_1_F_1_ plants produced many seeds, which were used for the genetic study.

### Genetic study of resistance and development of the resistant germplasm ANOM1

A population of 242 BC_1_F_2_ seeds was used for the genetic study. They were sown as described above, except for treating with gibberellic acid, and grown in pots under open-air conditions in the summer of 2017, resulting in 234 BC_1_F_2_ plants that reached maturity and could be evaluated for the presence/absence of broomrape parasitization. BC_1_F_3_ seeds from resistant BC_1_F_2_ plants were used to confirm the resistance and separation of homogenously resistant and segregating families. This was performed in pots under open-air conditions in the summer of 2019 using 9 individual BC_1_F_3_ plants per family, following the same procedures as in the BC_1_F_2_ evaluation. After a preliminary genetic mapping analysis, 10 BC_1_F_3_ families for which it was detected a discrepancy between the expected phenotype based on the genotypic class inferred from marker data and the results observed in the phenotypic evaluation of resistance were evaluated again for resistance/susceptibility to broomrape race G in the summer of 2021 using additional 12 BC_1_F_3_ plants per family and the same procedures as in the initial evaluation. BC_1_F_4_ plants from 6 resistant BC_1_F_3_ families, selected for visual resemblance to cultivated sunflower, seed production, and seed size, were also grown in 2021 to form the germplasm ANOM1 after confirmation of broomrape resistance at the individual plant level.

In all cases, the susceptible parent P21 and the resistant line DEB2 were used as controls. In all evaluations, sunflower plants were classified as resistant if they showed no emerged broomrape shoots and susceptible if they showed at least one emerged broomrape shoot. This is a common procedure in sunflower evaluation for broomrape resistance since the trait is commonly controlled by alleles at loci with major effects that determine the absence of emergence of broomrape shoots (Škorić et al. 2021).

### Allelic crosses

Allelic crosses between ANOM1 and the genotype DEB2 previously developed by our research group (Velasco et al. 2012) and carrying the gene *Or*_*Deb2*_ which also confers resistance to race G of broomrape (Velasco et al. 2012; Fernández-Aparicio et al. 2022) were carried out. A total of 3018 F_*2*_ plants coming from 27 F_1_ plants from the allelic cross between ANOM1 and DEB2 were grown in 2021 and 2022 in pots under open-air conditions and evaluated for race G (population GT) resistance as described above.

### Performance of the new germplasm ANOM1 against other broomrape populations

The germplasm ANOM1 was also evaluated with sunflower broomrape populations representative of the two races currently present in the Guadalquivir Valley area, where the research was conducted. They were broomrape population SP, a conventional race-F population of the Guadalquivir Valley gene pool (Martín-Sanz et al. 2016), and population IN180, collected in 2016 in Villanueva del Rey (Seville Province), and classified as race G. The evaluation was conducted in pots in winter 2021–2022 in the greenhouse and in summer 2022 under open-air conditions using sunflower lines NR5 and DEB2 (Martín-Sanz et al. 2016) as controls and 16 individual plants for each combination of broomrape population and sunflower germplasm. Procedures for soil preparation and inoculation were the same as described above.

### Tissue collection, DNA extraction, plant genotyping and gene mapping

Two fully expanded young leaves from each of the 234 BC_1_F_2_ plants of the mapping population were cut and frozen at −80 °C to be used for gDNA extraction. The leaf tissue was lyophilized and ground in a laboratory mill. gDNA was extracted as described in Pérez-Vich et al. (2004).

A set of 192 SNP markers developed and mapped by Bachlava et al. (2012) and Bowers et al. (2012), identified by SFW prefixes and evenly distributed across the 17 sunflower chromosomes, was genotyped in the mapping population using competitive allele-specific PCR assays based on KASP™ technology (LGC genomics, Teddington, Middlesex, UK). In an initial genetic linkage analysis, only polymorphic markers from Chr 4 were linked to the resistance gene. Based on these results, we additionally genotyped the mapping population with 7 SNP-AXIOM markers (AX-prefix) closely linked to broomrape resistance gene *Or*_*Deb2*_, previously mapped in Chr 4 (Fernández-Aparicio et al. 2022), and a set of SNP markers developed for *Or*_*Deb2*_ fine mapping (prefix Iasnip in the interval from 17 to 47) (Table S1, listed those that proved effective in the course of this research), using the same approach described above. Also, we developed as described below, based on ANOM1 and P21 cloned fragments, new SNP markers (prefix Iasnip in the interval from 95 to 106) (Table S1, listed those that proved effective in the course of this research) in the interval delimited by the initial genetic linkage analysis and used them to genotype the mapping population.

Genetic linkage analysis was performed using MAPMAKER v3.0 (Whitehead Institute, Cambridge, MA; Lander et al. 1987) using segregation data for all the SNP markers and the genotypic score of the individuals of the mapping population for the putative locus underlying resistance to broomrape. The genotypes for the putative *Or* locus were inferred from their corresponding phenotypes based on the evaluation of the BC_1_F_2_ and BC_1_F_3_ plants as follows. BC_1_F_2_ plants were scored as homozygous dominant for the putative *Or* locus if they were resistant and showed uniformly resistant plants in their respective BC_1_F_3_ progeny, heterozygous if they were resistant and their BC_1_F_3_ progeny segregated (i.e. showed both resistant and susceptible plants) and homozygous recessive if they were susceptible. Twelve resistant BC_1_F_2_ plants did not produce sufficient seeds for progeny evaluation, and they were scored as homozygous dominant or heterozygous (dominant score). Two-point analysis was used to group the marker loci. A LOD threshold of 15 and a maximum recombination fraction of 0.3 were used as linkage criteria. Three-point and multi-point analyses were used to determine the order and interval distances between the markers. Recombination fractions were converted to centiMorgans (cM) using the Kosambi mapping function. Linkage group maps were drawn using the MapChart software (Voorrips 2002).

### SNP marker development based on the sequence of P21 and ANOM1 cloned fragments

For the development of SNP markers Iasnip from 95 to 106, the genomic region delimited by the closest SNP markers identified in the initial genetic linkage analysis was extracted from the HanXRQr2.0-SUNRISE reference sunflower genome sequence (https://www.heliagene.org/HanXRQr2.0-SUNRISE) and used to design primers for amplification of about 800 to 1000 bp regions in P21 and ANOM1. Fifty-seven PCR primer pairs were designed with Primer3 (Untergasser et al. 2012) from protein-coding and genomic regions in the extracted sequence. PCR reactions were performed in 50 µl using 0.75 units of MyTaq DNA polymerase (Bioline, Meridian Life Science, Memphis, USA), 1 × MyTaq reaction buffer (containing 5 mM dNTPs and 15 mM MgCl_2_), 0.4 µM of each primer, and 100 ng of genomic DNA. The amplification was performed in a GeneAmp PCR system 9700 (Applied Biosystems, Foster City, CA, USA) with an initial denaturation step of 3 min at 95 °C followed by 34 cycles of 30 s at 95 °C, 30 s at annealing temperature (48–58 °C) and 1 min/kb at 72 °C, followed by an extension step of 20 min at 72 °C to create the poly-A tail. PCR fragments from 3 primers pairs (Table S1) which showed clear amplification in both genotypes were purified by means of the MEGAquick-spin Plus Total Fragment DNA Purification Kit (iNtRON Biotechnology, Inc., Korea), cloned into the pSpark TA Done DNA cloning vector as described by the manufacturer (Canvax Biotech SL, Córdoba, Spain), and Sanger sequenced (Stab Vida, Lisbon, Portugal). Sequences and their identities were confirmed using the BLAST software. Sequence alignments for each PCR product in both genotypes were carried out and used to group the sequences and to identify clusters corresponding to different loci since the fragment analysed has been described to contain duplicated regions (Fernández-Aparicio et al. 2022). Sequences from the same locus were analysed for SNP-DNA polymorphisms and 8 SNP markers were developed (Table S1). Sequence analysis was conducted using the Lasergene SeqMan Ultra and MegAlign Pro software within the DNASTAR, Inc. package.

### Characterization of the genomic region containing the putative *Or* resistance gene

All molecular markers linked to the resistance gene were mapped against the HanXRQr2.0-SUNRISE reference sunflower genome sequence (https://www.heliagene.org/HanXRQr2.0-SUNRISE). After their positions were determined, the annotated genes and their structure in the genomic region delimited by the recombinant SNP markers closest to the resistance gene were examined. In parallel, the chromosome-level genome assembly of *H. anomalus* was also used to analyse the sequence and structure of the protein-coding genes in the target region, delimited by the closest recombinant SNP markers. The *H. anomalus* genome sequence and assembly, derived from the accession ANO-2822 (Ames 32,950), was produced at UBC (University of British Columbia, Vancouver, Canada) and annotated at LIPMe-INRAe (Toulouse, France) using protocols and tools described in Badouin et al. (2017). This *H. anomalus* assembly, referred to as HanomANO2822-UBC throughout this manuscript, was submitted to NCBI on 11 September 2023 under SUBID: SUB13720851, BioProject: PRJNA1000454, and BioSample: SAMN36768292 and finally deposited at DDBJ/ENA/GenBank under the accession JAWUZI000000000. The version used in this manuscript is JAWUZI010000000. Target region genome sequence in *H. anomalus* HanomANO2822-UBC assembly was compared to that in *H. annuus*-HanXRQr2.0-SUNRISE reference sunflower genome sequence and also to itself (in order to test for duplications) using Dottup [EMBOSS (European Molecular Biology Open Software Suite) package; Rice et al. 2000]. In order to determine collinearity in the target region, tBlastn was used to compare the annotated proteins from *H. annuus*-HanXRQr2.0-SUNRISE against the genomic sequence in the *H. anomalus* HanomANO2822-UBC assembly. Also, sequence identities between proteins in the target region of both HanXRQr2.0-SUNRISE and HanomANO2822-UBC assemblies were obtained by BlastP. Gene orthologue inferences were also confirmed by identifying orthogroups between annotated protein coding genes in HanXRQr2.0-SUNRISE and *H. anomalus* HanomANO2822-UBC using OrthoFinder (Emms et al. 2019). Finally, for specific HanomANO2822-UBC annotated proteins, detailed in the results section, BlastP searches against the NCBI protein database were carried out and their protein domains were analysed by InterProScan (https://www.ebi.ac.uk/interpro/) and ProSite (https://prosite.expasy.org/). Also, multiple sequence alignments were generated by using the Clustal Omega programme using Lasergene MegAlign Pro software included in the DNASTAR, Inc. package.

### Characterization of resistance mechanisms

#### Rhizotron experiments

Two separate experiments were conducted, in both cases using six rhizotrons of each of the sunflower genotypes ANOM1 and the susceptible control B117 (Martín-Sanz et al. 2016). The rhizotrons consisted of two plates of Plexiglas 12 × 12 cm and were prepared following Louarn et al. (2016) and Le Ru et al. (2021) procedures with slight modifications. Sunflower seeds were sterilized in 4.8% sodium hypochlorite for 10 min and rinsed with sterile distilled water thrice. Sterilized sunflower seeds were sown in a mixture of sand/vermiculite (1:1 by volume) and maintained in a growth chamber at a constant temperature of 22 °C, 60% humidity, and 16 h photoperiod. Seeds of sunflower broomrape population GT were surface sterilized in 3.2% sodium hypochlorite with 0.001% Triton X-100 for 5 min and rinsed 3 times with sterilized distilled water using a 40 µm sterile cell strainer. Broomrape seeds were conditioned for six days in sterilized distilled water (3.3 mg seeds/ml) at 22 °C in the dark. Sunflower seedlings were transferred to glass fibre paper (reference 036294B, Dutscher, Bernolsheim, France) and inoculated with 3 ml of conditioned sunflower broomrape seeds, resulting in 10 mg of seeds per rhizotron. The rhizotrons were placed in a growth chamber at 22 °C 60% humidity and a 16-h photoperiod. They were watered three times per week with half-strength Long Ashton solution (Hewitt 1966) containing 370 µM phosphate. The roots were examined at 14, 21, 28, and 35 days post-inoculation (dpi) under a binocular microscope to determine the total number and stage of development of attachments to sunflower roots and the number of necrotic attachments. The stage of development of the attachments was based on the classification proposed by Martín-Sanz et al. (2016), with some modifications. The developmental stages considered were: T0: absence of attachment; T1: the attachment is formed but not actual tubercle yet visible; T2: tubercle with a diameter smaller than 1 mm; T3: tubercle with a diameter bigger than 1 mm and no visible stem buds; T4: tubercle with stem buds already formed or early stages of stem growth. The predominant stage of the attachments was recorded at each date. The percentage of germination of broomrape seeds was counted at 14 dpi.

Data were analysed using ANOVA for the variables: percentage of germination, number of attachments, and percentage of necrotic attachments. Arcsin transformation was previously applied to the data expressed in percentages (Sokal and Rolf 1995). Analyses were conducted separately for each evaluation date using the genotype and the experiment as fixed factors. If the interaction between genotype and experiment was significant, the two experiments were analysed separately.

### Histopathological study

Ten sunflower root pieces with *O. cumana* seedlings attached were cut from the sunflower plants of the rhizotron assay at 14, 21, 28, and 35 dpi using a binocular microscope. Samples were prepared as in Chabaud et al. (2022). Half of the samples were fixed in ethanol: acetic acid (3:1 by volume) for 10 min under vacuum, cleared in chloral hydrate 5 g/ml for 48 h under agitation and visualized with an Axioplan 2 light microscope (Zeiss, Jena, Germany). The remaining samples were fixed in FAA solution (10% formaldehyde, 5% acetic acid, and 50% ethanol) for 5 min under vacuum, dehydrated in alcohol series, and embedded in Technovit 7100 resin (Heraeus Kulzer, Germany). Thin sections of 10 µm were then made using a Reichert-Jung 2040 microtome (Leica Biosystems, Nussloch, Germany), stained with 0.2% toluidine blue for 3 min, mounted in DePeX mounting medium and scanned using a NanoZoomer image scanner (Hamamatsu Photonics, Japan). For the detection of phenolic compounds, hand-cut sections (with a razor blade) obtained from fresh root samples at 14, 21, 28, and 35 dpi were observed under epifluorescence (340–380 nm), as described in Lozano-Baena et al. (2007), in a Leica DM6 compound microscope with a Leica DFC7000 T digital camera (Wetzlar, Germany).

## Results

### Inheritance study

P21 parental line was uniformly susceptible (100% incidence) to broomrape population GT, with more than 10 broomrape stalks per plant in all cases. The F_1_ generation from the cross P21 x *H. anomalus* PI 468642 segregated for resistance to this broomrape population, with five susceptible and three resistant plants. Since the crosses were done using pollen from four *H. anomalus* plants indistinctly, because of the availability of very few small flowers, F_1_ segregation may indicate that the accession segregates for broomrape resistance, although the four plants used for the cross showed no emerged broomrape shoots. The BC_1_F_1_ generation from a backcross with one of the resistant F_1_ plants segregated into 12 resistant and 12 susceptible plants. This 1:1 ratio provided an initial indication that the resistance is controlled by dominant alleles at a single locus. This was confirmed in the analysis of the BC_1_F_2_ generation, which resulted in 187 resistant and 47 susceptible BC_1_F_2_ plants. These figures fit a 3:1 ratio (*χ*^2^ = 3.01; *P* = 0.08), suggesting that the resistance is dominant and controlled by a single locus. The evaluation of BC_1_F_3_ plants enabled the identification of two susceptible genotypes that had been tentatively classified as resistant in the BC_1_F_2_ evaluation, resulting therefore in a ratio of 185 resistant and 49 susceptible BC_1_F_2_ genotypes, which also fitted monogenic segregation (*χ*^2^ = 2.06; *P* = 0.15). The BC_1_F_3_ evaluation also permitted the separation of homozygotes and heterozygotes in 173 BC_1_F_2_ resistant genotypes, which revealed that 121 of the BC_1_F_3_ families segregated for resistance and the corresponding BC_1_F_2_ genotypes were heterozygotes for the resistance gene, whereas 52 BC_1_F_3_ families did not segregate for resistance and the corresponding BC_1_F_2_ genotypes were homozygotes. The results fitted the 2:1 ratio (heterozygotes: homozygotes) expected for a dominant gene (*χ*^2^ = 0.84; *P* = 0.36). The resistance locus has been named *Or*_*Anom1*_.

All 3018 F_2_ plants from the allelic cross between ANOM1 and DEB2 were resistant to broomrape population GT, indicating a linkage between the two *Or*_*Anom1*_ and *Or*_*Deb2*_ genes.

### Performance of ANOM1 germplasm against other broomrape populations

In addition to the race G sunflower broomrape population GT from Turkey, used for the development of the resistant germplasm ANOM1, this germplasm was also completely resistant to sunflower broomrape populations SP (race F) and IN180 (race G), both from the Guadalquivir Valley area in Southern Spain. For both broomrape populations, none of the plants of the ANOM1 germplasm and the resistant control DEB2 showed emerged broomrape shoots in the two evaluations conducted. On the contrary, all the plants of the susceptible control NR5 showed emerged broomrape shoots with both broomrape populations in the two evaluations.

### Genetic mapping of the *Or*_*Anom1*_ gene

**Fig. 1:**
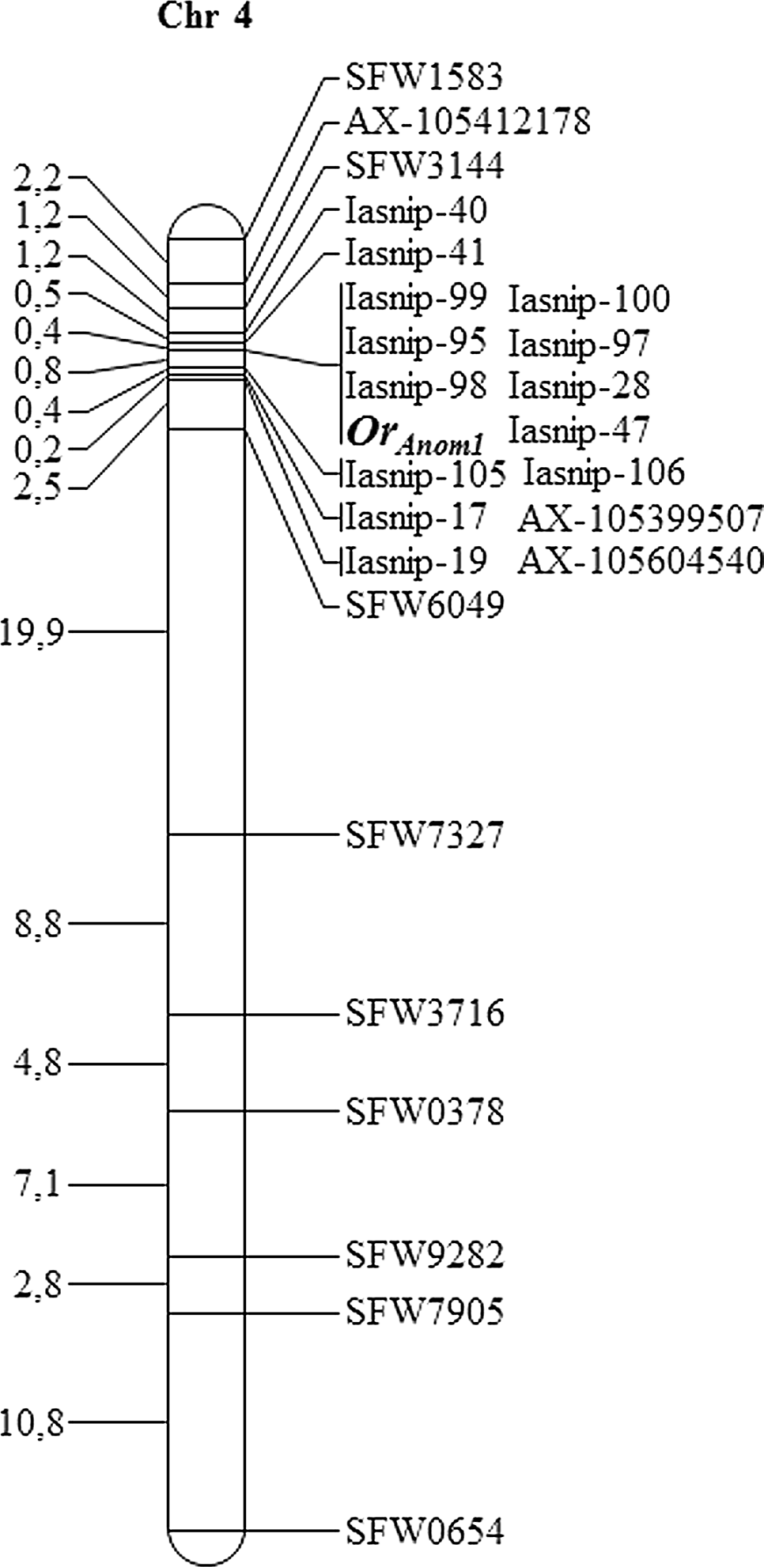
Linkage map of sunflower chromosome (Chr) 4 containing the *Or*_*Anom1*_ gene. The SFW prefix denote SNP marker loci mapped by Bowers et al. (2012); the AX prefix, SNP marker loci from the 600 k AXIOM® array developed at LIPME-INRAE (Toulouse, France); and the Iasnip prefix SNP markers developed by the authors. The interval distances in centiMorgans (cM) are shown at the left of the map

The *Or*_*Anom1*_ gene was mapped to Chr 4 of the sunflower genome (genotyping data provided in Table S2). It was located between SNP markers Iasnip-41 and Iasnip-105/Iasnip-106, which were 0.4 and 0.8 cM, respectively, from *Or*_*Anom1*_, and co-segregated with a total of 7 SNP markers (Fig. 1 and Table S3). The markers flanking *Or*_*Anom1*_ (Iasnip-41 and Iasnip-105/Iasnip-106) delineated a window of 1.32 Mb between physical positions 7,854,198 bp and 9,172,132 bp of the HanXRQr2.0 sunflower genome (https://www.heliagene.org/HanXRQr2.0-SUNRISE) (Table S3), which was almost completely coincident with that recently described on Chr 4 containing the wild-*H. debilis* derived *Or*_*Deb2*_ gene (from 7,892,288 bp to 9,272,600 bp of the HanXRQr2.0 assembly, Fernández-Aparicio et al. 2022). In the HanXRQr2.0 assembly, this region harbours a cluster of genes encoding RLPs (annotated as putative non-specific serine/threonine protein), and RLKs (annotated as putative protein kinases of the RLK-Pelle class, according to RLK class classification in Shiu and Bleecker 2001).

### Characterization of the *Or*_*Anom1*_ locus structure in the wild *H. anomalus* genome and identification of putative gene candidates

All molecular markers linked to *Or*_*Anom1*_ (Fig. 1) were mapped against the *H. anomalus* genome assembly (HanomANO2822-UBC) and their positions were determined (Table S3). Marker order between HanXRQr2.0 and HanomANO2822-UBC assemblies was overall conserved (Table S3), although HanXRQr2.0 Chr 4 was re-shuffled in HanomANO2822-UBC. Markers on top of HanXRQr2.0 Chr 4 mapped to Chr 7 in *H. anomalus* (Table S3), which is made up of part of Chr 4 and Chr 7 of *H. annuus* (Table S3), as previously reported for other wild *Helianthus* species (Barb et al. 2014; Ostevik et al. 2020). The *Or*_*Anom1*_ flanking markers Iasnip-41 and Iasnip-105 were mapped against the HanomANO2822-UBC genome sequence. Iasnip-105 located at position 10,352,424 bp in *H. anomalus* Chr 7, but Iasnip-41 was not found in this assembly. The closest marker on the left of Iasnip-41 (Iasnip-40) was located in the *H. anomalus* genome at position 9,087,369 bp in Chr 7 (Table S3). These two markers, Iasnip-40 and Iasnip-105, delineated a window of 1.27 Mb for the *Or*_*Anom1*_ locus in the *H. anomalus* genome. A dot plot analysis between the HanXRQr2.0 and HanomANO2822-UBC assemblies in the Iasnip-40 to Iasnip-105 region showed that this genomic sequence was largely divergent between these two assemblies (Fig. S1A). Also, a dot plot analysis comparing the Iasnip-40 to Iasnip-105 genomic region against itself revealed that this region in the *H. anomalus* assembly contained less repeated and less low complexity regions than in HanXRQr2.0 (Fig. S1B and Fig. S1C).

**Fig. 2:**
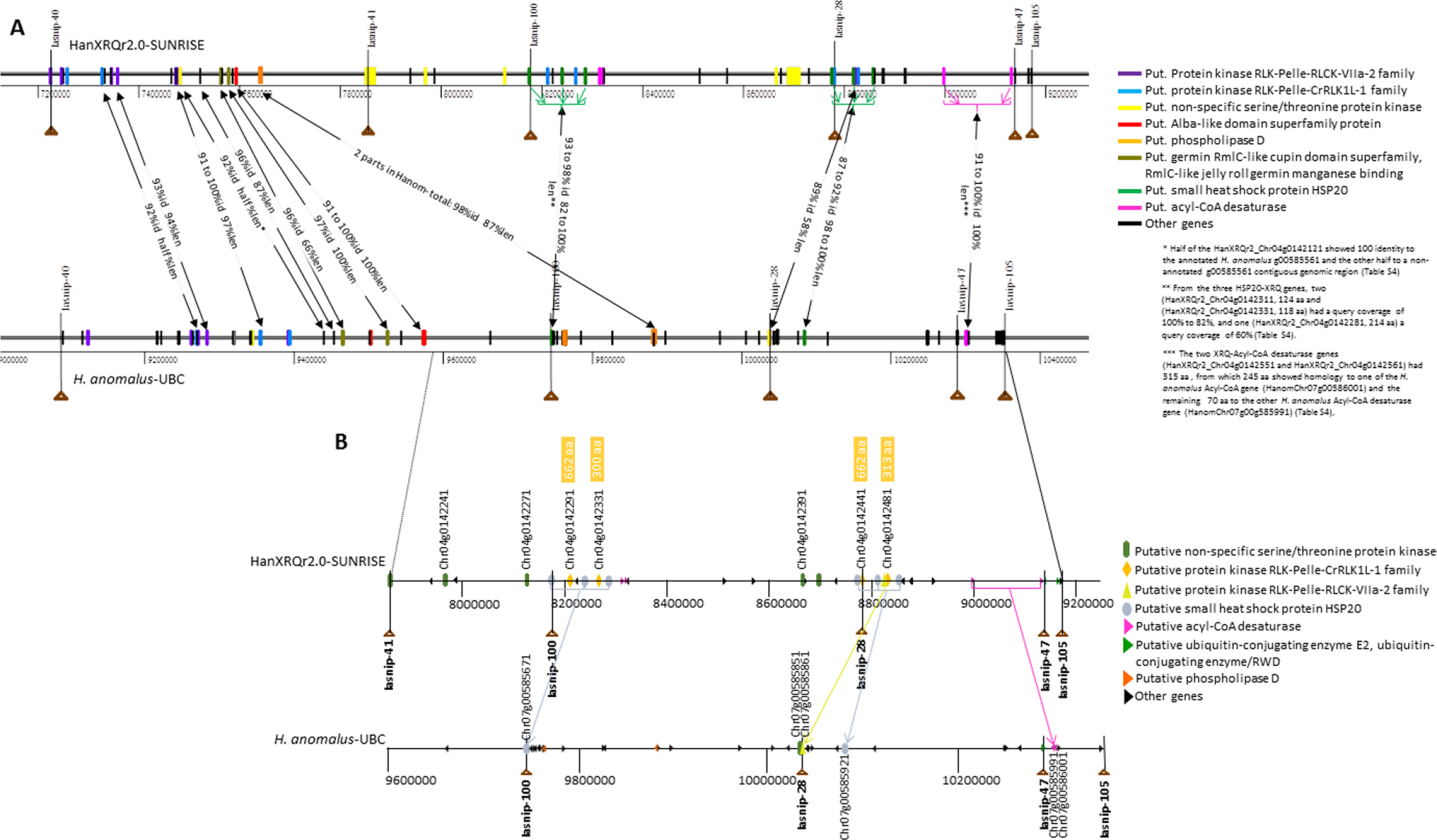
**a** Gene organization in the region delimited by SNP markers Iasnip-40 and Iasnip-105 in the *H. annuus* HanXRQr2.0 (top) and the *H. anomalus* HanomANO2822-UBC (bottom) assemblies. *H. annuus* and *H. anomalus* orthologous genes are associated by arrows, indicating levels of sequence identity (id), and matching length (len); **b** Gene organization in the region delimited by SNP markers Iasnip-41 (see text for the position of Iasnip-41 in *H. anomalus*) and Iasnip-105 in the *H. annuus* HanXRQr2.0 (top) and the *H. anomalus* HanomANO2822-UBC assemblies (bottom). *H. annuus* and *H. anomalus* orthologous genes are associated by arrows. Distances are indicated as bp

**Fig. 3:**
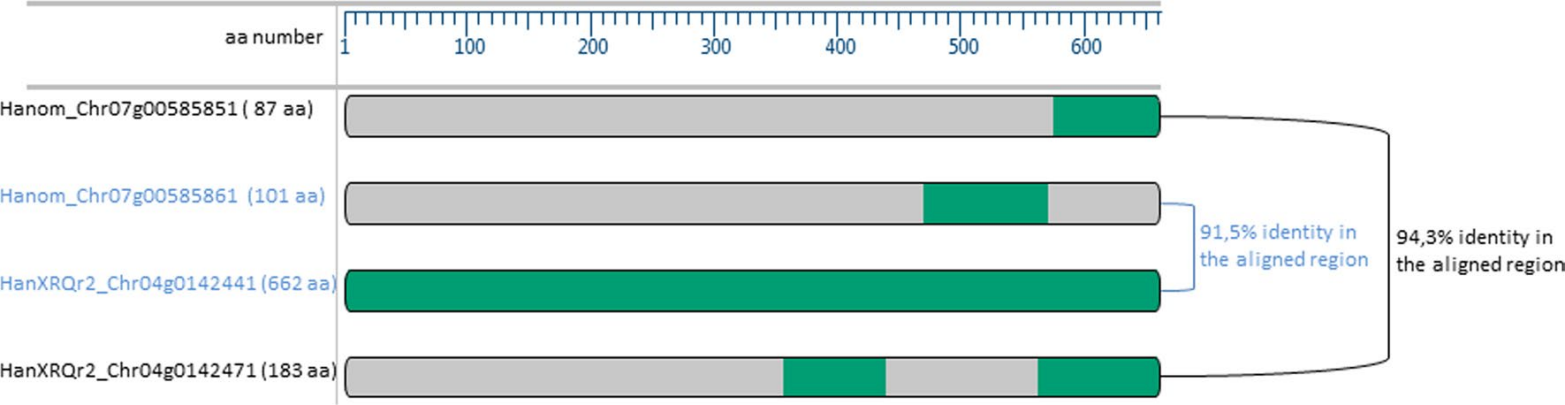
Schematic ClustalW alignment of protein kinase genes (*green colour*) HanomChr07g00585851 and HanomChr07g00585861 from *H. anomalus* HanomANO2822-UBC assembly*,* and HanXRQChr04g00142441 and HanXRQChr04g00142471 from *H. annuus* HanXRQr2.0 assembly. Similarity between closely related genes based on pairwise amino acid sequence comparisons is also indicated

The *H. anomalus* gene content and structure of the *Or*_*Anom1*_ locus in the 1.27 Mb region between markers Iasnip-40 and Iasnip-105 were determined and compared to those present in *H. annuus* HanXRQr2.0. Despite overall sequence differences observed between the HanXRQr2.0 and *H. anomalus* assemblies in this region (Fig. S1A), collinearity was found based on both (i) marker and (ii) high homologous protein coding gene (> 90% identity) order conservation (Fig. 2A and Table S4). The annotation of the *Or*_*Anom1*_ region revealed 71 protein-coding genes (Table S5). Within them, the most abundant (11 out of 71) were protein kinase genes. As Fernández-Aparicio et al. (2022) did for *Or*_*Deb2*_, detailed analysis of this protein kinase cluster was carried out due to (i) their abundance in this region, (ii) the fact that the only gene conferring resistance to *O. cumana* cloned to date is a receptor-like kinase (Duriez et al. 2019), and (iii) their essential role in several plant-pathogen interactions (Dievart et al. 2020). The present analysis revealed the absence of LRR-RLPs (annotated as non-specific serine/threonine protein kinases) in *H. anomalus*-*Or*_*Anom1*_. The only two *H. anomalus* genes annotated as non-specific serine/threonine protein kinases (HanomChr07g00585521 and HanomChr07g00585851, Table S5) showed indeed high similarity (100% and 94.3% identity, respectively) to a part of protein kinases of the RLK-Pelle class (HanXRQ2Chr04g00141991 and HanXRQ2Chr04g00142471, respectively) (Table S5 and Fig. 3 for HanomChr07g00585851). Therefore, the only group of kinases present in the *H. anomalus*-*Or*_*Anom1*_ locus was constituted by those of the RLK-Pelle class. Of these, HanomChr07g00585341 and HanomChr07g00585531 (with 588 and 691 aa, respectively) had two kinase domains, whereas the rest had only one (Fig. S2). Best BlastP hits of the Iasnip-40 to Iasnip-105-HanomANO2822-UBC kinases against the NCBI protein database revealed orthologous genes in HanXRQr2.0 with > 90% sequence identity and query coverage at the target Iasnip-40 to Iasnip-105 interval (6 genes from 9.3 to 10.0 Mb in HanomANO2822-UBC with two groups: (i) HanomChr07g00585521 to HanomChr07g00585551 showing homology to HanXRQ2Chr04g00141991 and HanXRQ2Chr04g00142101, and (ii) HanomChr07g00585851 and HanomChr07g00585861 with homology to HanXRQ2Chr04g00142471 and HanXRQ2Chr04g00142441, respectively) (Table S5), or at a left-side adjacent interval (5 genes from 9.1 to 9.28 Mb in HanomANO2822-UBC) (Table S5).

As indicated above, the *Or*_*Anom1*_ left closest marker (Iasnip-41), which showed two recombinants with *Or*_*Anom1*_, revealed no hits in *H. anomalus* HanomANO2822-UBC genome assembly. However, these Iasnip-41-*Or*_*Anom1*_ recombinants together with the collinearity found in the *Or*_*Anom1*_ region, with overall marker and gene order conservation (Fig. 2A), and the positions of markers Iasnip-28, 47 and 100 co-segregating with *Or*_*Anom1*_, suggested a more delimited Chr 7 *H. anomalus* region for *Or*_*Anom1*_ (Fig. 2B), which comprised 37 genes (Fig. 2B and Table S5). Among these, there were *H. annuus*-XRQ orthologous genes such as small heat shock protein HSP20 (HanomChr07g00585671 and HanomChr07g00585921), phospholipase D (HanomChr07g00585731 and HanomChr07g00585791), acyl-CoA desaturase (HanomChr07g00585991 and HanomChr07g00586001) and a kinase belonging to the RLCK-VIIa-2 family (HanomChr07g00585851) (Fig. 2A, B). A putative transcription factor of the C2H2 family (HanomChr07g00585881) was also identified (Table S5), which was not found in HanXRQr2.0.

Within this more delimited region, only two kinase genes were identified (Table S5): (i) the orthologue mentioned above HanomChr07g00585851, annotated as a non-specific serine/threonine protein kinase but similar (94.3% identity), as described above, to the HanXRQr2Chr04g00142471 protein kinase of the RLK-Pelle-RLCK-VIIa-2 family (Fig. 3), and (ii) HanomChr07g00585861 annotated as a RLK-Pelle class kinase belonging also to the RLCK-VIIa-2 family, which shared 91% identity with a part of HanXRQ2Chr04g00142441 [a RLK-Pelle class kinase belonging to the CrRLK1L-1 (*Catharanthus roseus* RLK-like)] (Fig. 3).

### Characterization of resistance mechanisms

The analysis of variance indicated that both the experiment (P < 0.05) and the interaction between the experiment and the genotype (P < 0.01) were significant for the percentage of germination of broomrape seeds. Accordingly, the two experiments were analysed separately. In the first experiment, seed germination averaged 85% on B117 and 89.5% on ANOM1, which were not significantly different (*P* > 0.05). In the second experiment, broomrape seeds showed significantly (*P* < 0.05) lower germination percentage on ANOM1 (77.29%) than on B117 (86.29%).

**Fig. 4:**
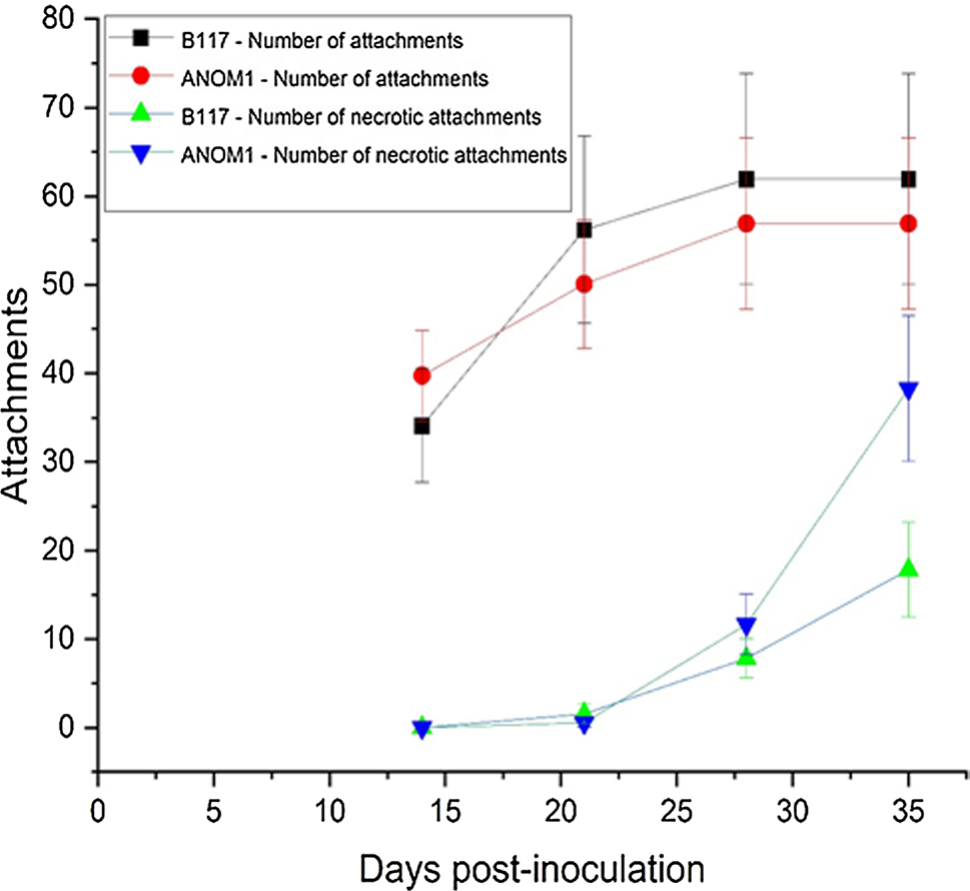
Number of total attachments and number of necrotic attachments of sunflower broomrape population GT in the roots of sunflower control genotype B117, susceptible to the broomrape population, and ANOM1, resistant to the broomrape population, evaluated in rhizotrons. Data are given as mean ± standard error

B117 and ANOM1 did not differ significantly for the total number of attachments on any evaluation dates (Table 1). The predominant stage of development of the attachments on B117 was T2 at 14 dpi, T3 at 21 dpi, and T4 at both 28 and 35 dpi (Fig. S3). Conversely, no progress of the attachments was observed on ANOM1; they reached the developmental stage T1 (broomrape attached to the sunflower root but no actual tubercle) at 14 dpi with no additional growth progress at 21, 28 and 35 dpi (Fig. S3). Instead, a high percentage of necrotic attachments, significantly higher (*P* < 0.01) than in B117, was observed in ANOM1 roots at 35 dpi. They averaged 28.78% for B117 and 67.25% for ANOM1 (Table 1; Fig. 4).

**Table 1:** Number of total attachments, number of necrotic attachments, percentage of necrotic attachments, and predominant stage of the attachments of sunflower broomrape population GT in the roots of sunflower control genotype B117, susceptible to the broomrape population, and ANOM1, resistant to the broomrape population, evaluated in rhizotrons from 14 to 35 days post-inoculation (dpi). Data are given as mean ± standard error, except for the percentage of necrotic attachments in which only the mean value is given^1^

DPI	Genotype	Attachments	Necrotic attachments	% Necrotic attachments	Predominant stage^2^
14	B117	34.09 ± 6.39	0.00	0.00	T2
	ANOM1	39.73 ± 5.08	0.00	0.00	T1
21	B117	56.18 ± 10.56	1.55 ± 1.11	2.76	T3
	ANOM1	50.09 ± 7.24	0.55 ± 0.45	1.10	T1
28	B117	61.91 ± 11.88	7.82 ± 2.21	12.63	T4
	ANOM1	56.91 ± 9.68	11.64 ± 3.41	20.45	T1
35	B117	61.91 ± 11.88	17.82 ± 5.32	28.78	T4
	ANOM1	56.91 ± 9.68	38.27 ± 8.21^*^	67.25^**^	T1

^1^Statistical significance of the differences between B117 and ANOM1 computed with ANOVA within each evaluation date. * = significant at *P* < 0.05; ** = significant at *P* < 0.01

^2^T0: absence of attachment; T1: the attachment is formed but not actual tubercle yet visible; T2: tubercle with a diameter smaller than 1 mm; T3: tubercle with a diameter bigger than 1 mm and no visible stem buds; T4: tubercle with stem buds already formed or early stages of stem growth

**Fig. 5:**
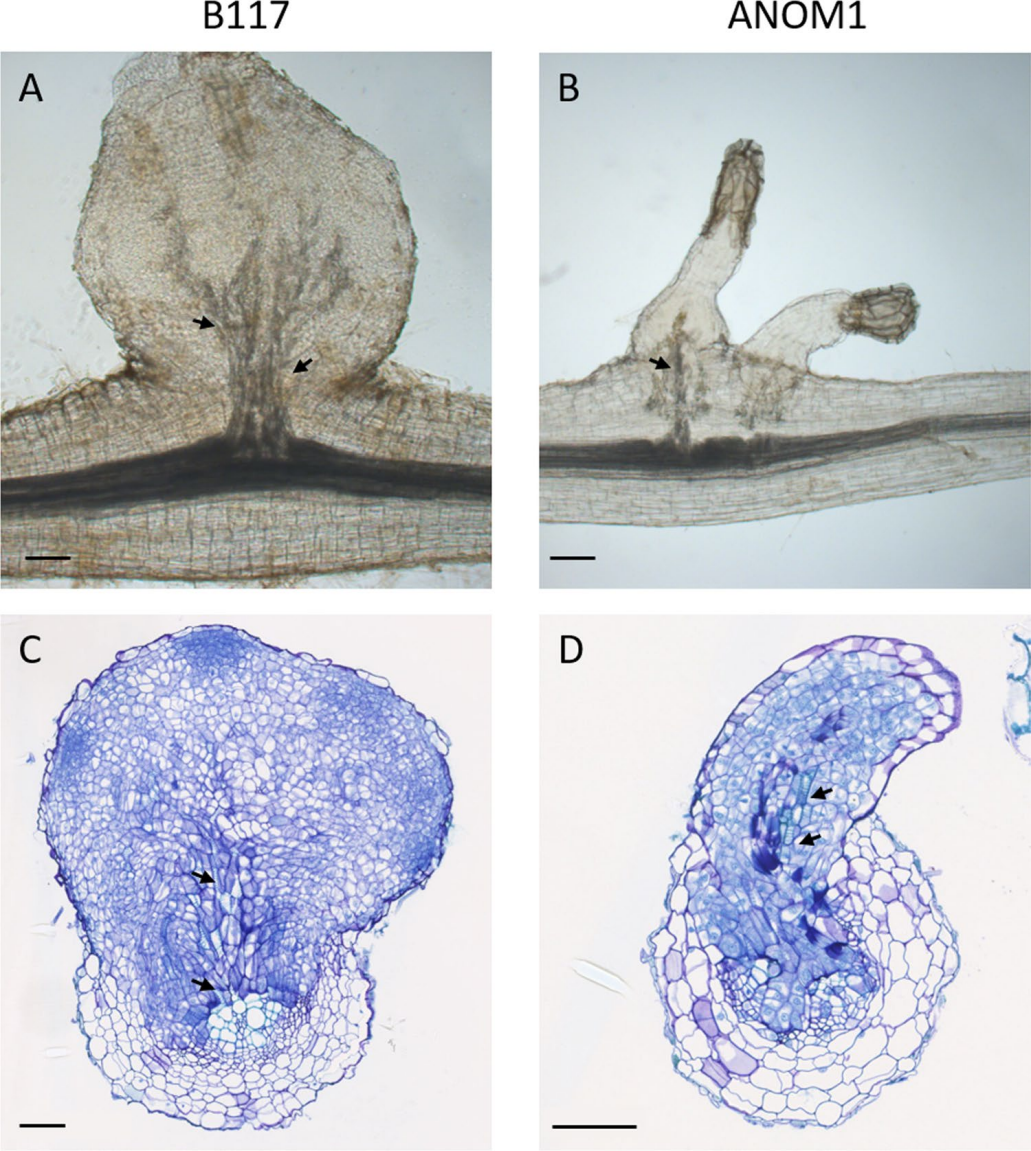
Vascular connection between *O. cumana* (population GT) and sunflower roots at 14dpi. Whole cleared samples of tubercles on **a** B117 and **b** ANOM1 roots. Xylem is stained dark grey. Transversal thin sections of a broomrape connected to **c** B117 and **d** ANOM1 roots stained with TBO. Xylem vessels stain light blue. *O. cumana* is connected to the sunflower root via xylem strands (arrows). Scale bars = 100 µm

**Fig. 6:**
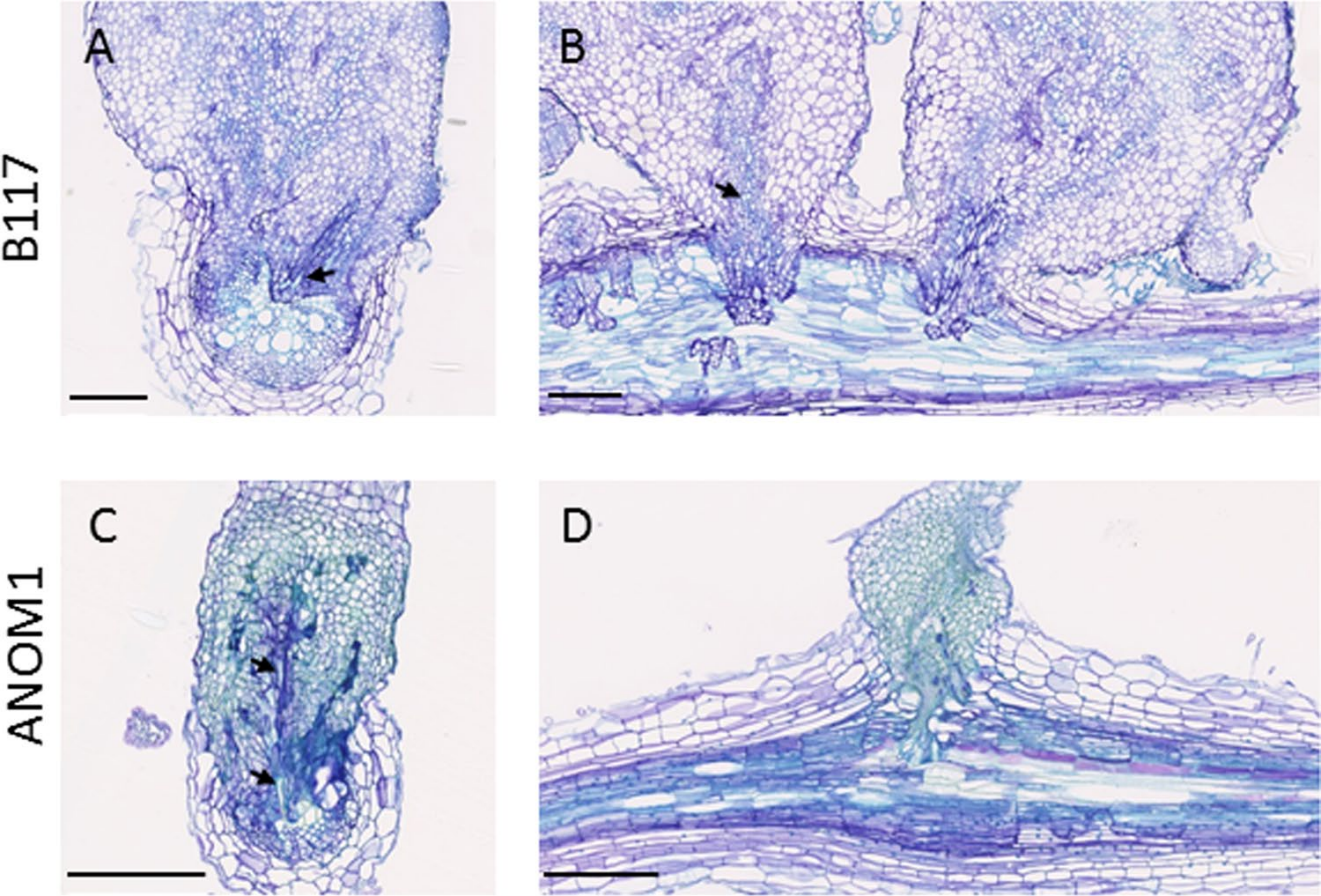
Differences between *O. cumana* (population GT) attached to resistant ANOM1 and susceptible B117 genotypes at 28dpi. **a** Transversal and **b** longitudinal thin sections of *O. cumana* attached to B117 stained with TBO. **c** Transversal and **d** longitudinal thin sections of *O. cumana* attached to ANOM1 stained with TBO. Xylem vessels stain light blue (arrows). Phenolic compounds accumulation stains green. Scale bars = 250 µm

**Fig. 7:**
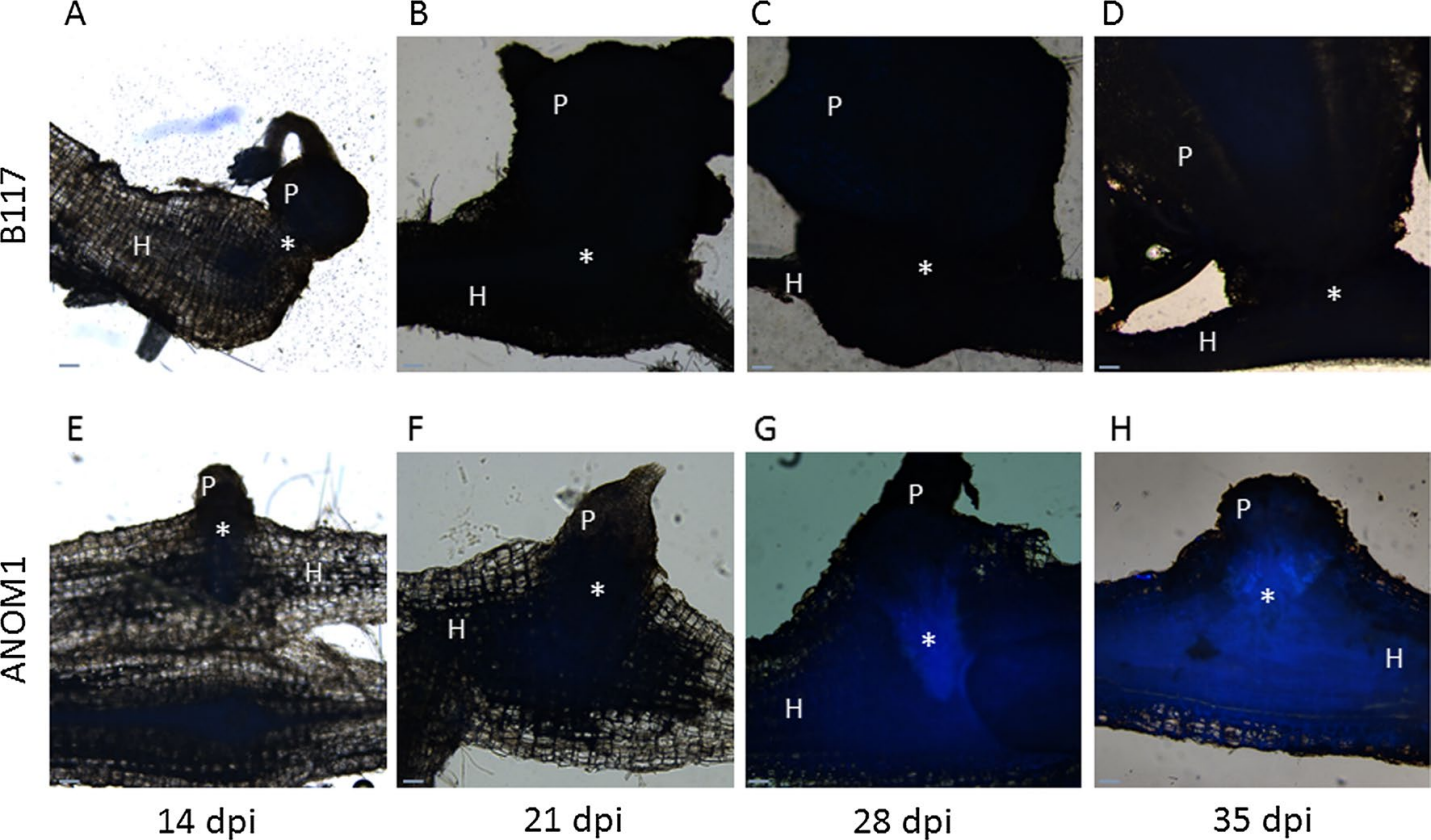
Fresh hand-cut sections of *O. cumana* (population GT) attached to sunflower roots observed under epifluorescence (340–380 nm). **a**–**d** Samples collected in the susceptible genotype B117 at 14, 21, 28 and 35 dpi, respectively. **e**–**h** Samples collected in the resistant genotype ANOM1 at 14, 21, 28 and 35 dpi, respectively. Phenolic compounds are detected as blue fluorescence. P, parasite; H, host; *, parasite-host connection. Scale bars = 100 µm

Histopathological studies were carried out on infected roots of ANOM1 and B117 to detail the resistant mechanism of ANOM1. In whole cleared samples at 14 dpi, xylem strands inside the parasite towards the host root were visible in both resistant and susceptible lines suggesting the connection to the host vascular system (Fig. 5). Cross thin sections revealed that *O. cumana* intrusive cells had reached the central cylinder of the sunflower root at 14 dpi and broomrape vessels were differentiated, corroborating the vascular connection with the host in samples of both lines (Fig. 5). This indicated that a post-attachment resistance mechanism operating at a later stage after vascular connections have been established was taking part in the ANOM1 resistant line. This vascular connection in ANOM1 was also clearly detected at 21dpi, 28dpi and 35 dpi, in both whole cleared samples and thin sections (Fig. S4). In thin sections, the samples taken from 28 dpi onwards were coloured green in the resistant phenotype ANOM1 after TBO staining (Fig. 6), as well as the host cells in proximity to the attachment, what indicated likely accumulation of phenolic compounds. This was also visible as brownish staining in whole cleared root samples (Fig. S4). To corroborate the phenolic compounds accumulation, fluorescence (340–380 nm) was measured in fresh hand cuts. A difference in fluorescence was observed between samples from both ANOM1 and B117 phenotypes. Also from 28 dpi onwards, fluorescence intensity was higher at the bottom of the broomrapes in contact with the ANOM1-resistant host root and absent in the broomrapes attached to the B117 susceptible line, which corroborated the presence of compounds of a phenolic nature in the resistant one (Fig. 7).

## Discussion

Sunflower crop wild relatives have been greatly beneficial for cultivated sunflower improvement, providing plant breeders with a diverse genetic pool of potentially useful traits (Seiler et al. 2017). Particularly, wild *Helianthus* species have represented a substantial reservoir of genes conferring resistance to broomrape (Seiler and Jan 2014). The genus *Helianthus* encompasses both annual and perennial species. Whereas most of the perennial species are immune to broomrape, resistance to this parasitic weed in annual species is less frequent, particularly to the most virulent races (Seiler and Jan 2014). However, for the annual species *H. anomalus*, accessions showing resistance to the Spanish race E (Ruso et al. 1996) and race F (Fernández-Martínez et al. 2000) of broomrape have been identified. For the more recent and virulent race G, Chabaud et al. (2022) identified one accession (PI 468638) of this species which showed resistance to a Romanian race G, although the number of plants evaluated was limited. In this study, accession PI 468638 was also resistant to a Turkish race G, together with another *H. anomalus* accession evaluated, PI 468642.

After the identification of wild *Helianthus* accessions resistant to broomrape, introgression of the resistance gene into the cultivated gene pool should be achieved by conventional crossing, backcrossing and selection for the resistance trait. Contrarily to perennial species, interspecific hybridization using annual wild *Helianthus* spp. is not particularly complex. For example, the successful development of race E and G resistant sunflower germplasm incorporating resistance genes from the wild annuals *H. deserticola* (Hladni et al. 2009), and *H. debilis* ssp. *tardiflorus* (Velasco et al. 2012), respectively, has been reported. Within this research, the race G-resistant germplasm ANOM1 was developed from accession PI 468642 after interspecific crossing, selection for dominant resistance, backcrossing, and visual resemblance to cultivated sunflower, seed production, and seed size.

Resistance to *Orobanche* spp. in crop plants is generally under polygenic, non-race specific control. Sunflower is a notable exception, where resistance has been found to be in most cases monogenic and dominant (Pérez-Vich et al. 2013). One single dominant gene controlling race G resistance has also been found in the germplasm derived from *H. anomalus* developed in this study, and the gene has been named *Or*_*Anom1*_. This monogenic and dominant inheritance is particularly valuable for F_1_ hybrid seed production, as the genetics is simple and the resistance gene is only introgressed in one of the parents. Since sunflower broomrape resistant hybrids are mainly based on single dominant *Or* genes, the development of new sources such as the one described in this study will facilitate the breeding progress. In addition, it will contribute to broaden the genetic basis of broomrape resistance in the cultivated sunflower pool.

To date, major broomrape resistance genes have been located on three sunflower chromosomes: Chr 3 (*Or5* and *or*_*ab-vl-8*_; Tang et al. 2003; Pérez-Vich et al. 2004; Imerovski et al. 2016, 2019), Chr 7 (*HaOr7*, Duriez et al. 2019) and Chr 4 (*Or*_*Deb2*_ and *Or*_*SII*_; Martín-Sanz et al. 2020; Fernández-Aparicio et al. 2022). The *Or*_*Anom1*_* gene* has also been mapped to Chr 4 in this study, and its linkage with the race G resistance gene *Or*_*Deb2*_ has also been confirmed with allelic crosses between genotypes ANOM1 and DEB2, carrying, respectively, each of the genes. In fact, both *Or*_*Anom1*_ and *Or*_*Deb2*_ genes have been located almost within the same genomic interval in Chr 4, *Or*_*Anom1*_ between physical positions 7,854,198 bp and 9,172,132 bp of the HanXRQr2.0 sunflower reference genome, and *Or*_*Deb2*_ from 7,892,288 bp to 9,272,600 bp (Fernández-Aparicio et al. 2022). Also, the *Or*_*SII*_ gene conferring post-haustorial resistance to races F and G is located at an *Or*_*Deb2*_ tightly linked position in Chr 4 (Martín-Sanz et al. 2020; Fernández-Aparicio et al. 2022). Therefore, it is clear from these studies that the Chr 4 *Or*_*Anom1*_ region harbours a broomrape resistance locus with different tightly liked genes or gene alleles originating from different wild relatives, including *H. anomalus* and *H. debilis*. It will have to be determined with further fine mapping studies if the genomic arrangement of *Or*_*Anom1*_ and *Or*_*Deb2*_ within this locus corresponds to a cluster of tightly linked resistance genes or it is a single gene with several alleles. Introgressed resistance genes from different crop wild relatives clustering to the same genomic region have also been described in sunflower for *Pl* genes conferring resistance to downy mildew [caused by the oomycete *Plasmopara halstedii* (Farl.) Berlese & de Toni]. For example, *Pl* genes introgressed from wild *H. annuus* and *H. tuberosus* have been found as tightly linked arrays clustering on Chr 4, tracing to wild *H. annuus* and *H. argophyllus* on Chr 8, and derived from wild *H. annuus*, *H. argophyllus* and *H. tuberosus* on Chr13 (summarized in Qi et al. 2023).

The gene structure and nature within the genomic *Or*_*Anom1*_ region, coincident with that of Chr 4 *Or*_*Deb2*_, in the reference sunflower genome sequence (obtained from the cultivated sunflower line XRQ), were described by Fernández-Aparicio et al. (2022). Since (i) it has been shown that resistance gene repertories vary at phylogenetic scales within genera and species (Steinbrenner 2020) and (ii), more specifically in sunflower, that introgressions from wild species not only enhance allelic diversity but also increase the total number of genes in the sunflower pan-genome so that the content of an introgression is not necessarily predictable from a reference sequence (Hübner et al. 2019), the *Or*_*Anom1*_ region was analysed in the recently obtained chromosome assembled genome sequence of *H. anomalus*. This species is derived from an ancient hybridization event likely involving ancestors of the *H. annuus* and *H. petiolaris* clades (Rieseberg 2006; Owens et al. 2023) and chromosomal rearrangements among them have been described (Lai et al. 2005). In fact, we have seen that markers on top of HanXRQr2.0 Chr 4 mapped to Chr 7 in *H. anomalus*, which is made up of part of the *H. annuus* Chr 4 and of the *H. annuus* Chr 7 (Table S3). Rearrangements between Chr 4 and Chr 7 have already been reported in wild sunflowers (Barb et al. 2014; Ostevik et al. 2020). Actually, the Chr 4-Chr 7 configuration of *H. anomalus* is ancestral in the genus *Helianthus* and already present in ancestral reconstructed specific karyotypes from a subsection of annual sunflowers (Ostevik et al. 2020). Despite this rearrangement, marker order was overall conserved in *H. anomalus*, taking into account the physical positions of the markers in the *H. anomalus* genome assembly (Table S3). Therefore, the *Or*_*Anom1*_ locus was found on top of Chr 7 in the HanomANO2822-UBC assembly.

The putative gene candidates identified in the *Or*_*Anom1*_ target region raised the question if any of them may underlie this gene. Within this region, Fernández-Aparicio et al. (2022) pointed to nine RLK and RLP genes as being the best candidates for *Or*_*Deb*2_ in the reference genome of cultivated sunflower (HanXRQr2.0-SUNRISE). This was supported by their abundance in this region, the fact that the only gene conferring resistance to *O. cumana* cloned to date is a receptor-like kinase (Duriez et al. 2019), and the essential role of these kind of genes in different biotic stress responses in plants (Wang et al. 2008; Dievart et al. 2020). In this study, and since *Or*_*Anom1*_ was mapped to the same *Or*_*Deb*2_ interval, these genes were also considered the best *Or*_*Anom1*_ candidates, and their nature and structure in the wild *H. anomalus* genome were analysed. In this species, RLPs were not found in the *Or*_*Anom1*_ target region, leaving RLKs as the only putative candidate for *Or*_*Anom1*_. These protein kinases were RLK-Pelle class, having one or two-fused kinase domains, and no other domains. Kinase-only disease resistance genes include, for example, the tomato *Pto* gene determining resistance to the plant pathogenic bacterium *Pseudomonas syringae* pv *tomato* (Ntoukakis et al. 2014). Dual-kinase genes show structural similarities to tandem kinase-pseudokinase genes underlying the barley *Rpg1* and the wheat *WTK1* (*Yr15*), *WTK2* (*Sr60*), *WTK3* (*Pm24*), and *WTK4* genes which determine resistance to biotrophic fungal pathogens (Klymiuk et al. 2021; Fahima and Coaker 2023).

Although the left *Or*_*Anom1*_ recombinant marker (Iasnip-41) was not found in the *H. anomalus* HanomANO2822-UBC genome assembly, there were different reasons detailed in the results sections suggesting a more delimited Chr 7 *H. anomalus* region for *Or*_*Anom1*_, which comprised 37 genes (Table S5). Among them, only two RLKs were identified: (i) HanomChr07g00585851 (87 aa) with no domain hits in ProSite and similar to the HanXRQr2.0 Chr 4 orthologue Chr04g00142471 (protein kinase of the RLK-Pelle-RLCK-VIIa-2 family) (Fig. 3) and (ii) HanomChr07g00585861 (101 aa) having one kinase domain and sharing 91% identity with a part of HanXRQ2Chr04g00142441 [RLK-Pelle class kinase belonging to the CrRLK1L-1 (*Catharanthus roseus* RLK1-like)]. By examining matching regions of these two RLK *H. anomalus* genes with their XRQ2.0 homologues, they were found in fact to match contiguously to the longer orthologue HanXRQ2Chr04g00142441 (Fig. 3). This kind of gene rearrangement generating split and shorter (or contrarily fused and longer) RLKs has already been described for clustered RLKs and has been related to localized gene duplications and/or transposon element insertions (Shiu et al. 2004; Zhang et al. 2005). However, local misassembles and annotation errors cannot be completely excluded. On the other hand, other protein-coding genes which have been associated with pathogen resistance in plants found in the *Or*_*Anom1*_ limited target interval might also be considered as *Or*_*Anom1*_ candidates. These included a putative transcription factor of the C2H2 family (HanomChr07g00585881) and an ubiquitin-conjugating enzyme E2 (HanomChr07g00585971). InterPro analyses of the HanomChr07g00585881 amino acid sequence revealed a RING (really interesting new gene)-type zinc finger domain, which is involved in mediating protein–protein interactions. A general function of this RING domain is likely to be an E3 ubiquitin-protein ligase activity (Freemont 2000). The ubiquitin-conjugating enzyme (E2) and ubiquitin ligase enzyme (E3) are components of the ubiquitin–proteasome system (UPS), together with ubiquitin, ubiquitin-activating enzyme (E1) and 26S proteasome (Sadanandom et al. 2012). This UPS system is one of the main pathways for post-translational protein modifications, affecting many cellular processes (Sadanandom et al. 2012) and its role in the regulation of plant defence against pathogens has been described (Devoto et al. 2003; Craig et al. 2009). Particularly, ubiquitin-conjugating enzyme (E2) and ubiquitin ligase enzyme (E3) have been associated with disease resistance in crops such as rice (Ishikawa et al. 2014; Wang et al. 2015; Liu et al. 2021) or tomato (Zhou et al. 2017). It has to be mentioned that HanomChr07g00585881 sunflower orthologous genes found through NCBI blast were not located on Chr 4, but on sunflower Chr 2 [100% coverage, 95.2% identity; RHA438 assembly (Huang et al. 2023)] and 5 [85% coverage, 91.9% to 93.6% identity; RHA348, HA300, OQP8, LR1, PI659440 (Huang et al. 2023) and XRQr2.0 assemblies]. Regarding HanomChr07g00585971, its orthologous gene HanXRQr2_Chr04g0142581 was located in the sunflower Chr 4 *Or*_*Anom1*_ limited target interval. Due to the synteny observed between *H. annuus* and *H. anomalus* in this interval and the possible existence of local misassembles, HanomChr07g00585881 might be considered a less robust candidate than the others. Further *Or*_*Anom1*_ fine mapping and the genome sequence of ANOM1 would be necessary to discern among the different hypotheses regarding the nature of the functional *Or*_*Anom1*_ gene.

Gaining insights into the sunflower mechanisms of resistance to broomrape is not only essential for understanding the underlying molecular processes involved, but also for designing physiology-based resistance breeding strategies (Pérez-Vich et al. 2013). Different resistance strategies to prevent parasite infection have been documented, each one targeting one stage of the parasite life cycle. Resistance mechanisms at the initial stage of the host–parasite interaction have been described in sunflower genotypes that exhibit reduced release of germination stimulants, as well as those that release germination inhibitors (Labrousse et al. 2001; Serghini et al. 2001). Our data showed the resistance conferred by *Or*_*Anom1*_ is not related to *O. cumana* germination. Post-attachment mechanisms operating in a first step preventing the establishment of effective vascular connections between sunflower and *O. cumana* have been identified for the major genes *HaOr7* (Duriez et al. 2019) and *Or*_*Deb2*_ (Fernández-Aparicio et al. 2022), which block *O. cumana* intrusion mainly at the cortex. Lignification, protein crosslinking, or suberization, which thicken in the host–parasite interface, have been described as mechanisms blocking *O. cumana* to reach the endodermis (Labrousse et al. 2001; Echevarría-Zomeño et al. 2006; Sisou et al. 2021; Chabaud et al. 2022). In our study, no physical barriers were observed impeding the penetration of the parasite into the root in the ANOM1 genotype. In fact, whole cleared samples and histological sections revealed the intrusion of the parasite until the central cylinder of the host root, where xylem vessels were differentiated and vascular connections were established, which differs from the mechanisms described for *HaOr7* and *Or*_*Deb2*_.

After vascular connections were formed, monitoring the development of the tubercles showed the first difference between the susceptible control and the ANOM1 resistant genotype. While tubercles attached to the susceptible B117 line never stopped growing, those attached to the ANOM1 line did not increase in size. Moreover, necrotic tubercles started to appear 21 dpi and by the end of the experiment more than 70% of the tubercles attached to the resistant line were dead. Although there were some tubercles attached to the resistant line that seemed healthy at 35 dpi, their arrested growth and the fact that the resistant plants showed no broomrape emergence from the soil when tested in pots suggested that these tubercles would most likely become necrotic before emerging from the ground. The susceptible line also presented necrotic tubercles, but in much less quantity. This can be explained by the high trophic competition between broomrapes due to the finite amount of assimilate available in each plant for all the parasites attached and the limited space where they grow inside the rhizotrons (Labrousse et al. 2001). Chabaud et al. (2022) evaluated a collection of wild *Helianthus* accessions for resistance mechanisms to *O. cumana*, in which unfortunately *H. anomalus* could not be evaluated in rhizotrons due to the poor germination ability of the accessions tested (M. Chabaud, personal communication). These authors described a group of wild species including *H. bolanderi*. *H. debilis*, *H. petiolaris*, and *H. praecox* which showed also tubercle necrosis revealing late resistance mechanisms.

Whole cleared samples, histological sections stained with TBO and fluorescence analysis showed phenolic accumulation from 28 dpi onwards in the ANOM1 genotype, revealing this as one of the main mechanisms for host defence reaction. This mechanism has also been described in other interactions with *Orobanche* spp. For example, Lozano-Baena et al. (2007) studied the interaction between *Medicago truncatula* and its parasite *Orobanche crenata* and reported a resistance mechanism after the vascular connection of the parasite in which phenolic compounds were released at the infection point, leading to the parasite’s cells death. In sunflower, Martín-Sanz et al. (2020) described a late post-attachment resistant mechanism conferred by the *Or*_*SII*_ gene and also observed phenolic compounds inside parasite tubercle cells and neighbouring host tissue, suggesting that these compounds produced toxicity and reduced the growth of the parasite structures. However, *Or*_*SII*_ and *Or*_*Anom1*_ resistance mechanisms differ in that *Or*_*SII*_ did not produce necrotic attachments in rhizotron studies and allowed tubercle development, being resistance mechanism observed at a very late stage of the broomrape development as underdeveloped broomrape shoots with reduced growth even after broomrape shoot emergence (Martín-Sanz et al. 2020), while in ANOM1 broomrape growth was stopped at early stages (mainly at T1) and most of them became necrotic.

In conclusion, a new monogenic and dominant resistance to race G of broomrape has been identified in the wild species *H. anomalus* and introgressed to sunflower. The resistance gene *Or*_*Anom1*_ was mapped to the RLK-RLP resistance locus on Chr 4, and *Or*_*Anom1*_-candidate genes were identified within the *H. anomalus* genome assembly. The Chr 4 RLK-RLP locus also harbours the *Or*_*Deb2*_ and *Or*_*SII*_ resistance genes. However, the different natures of the associated resistance mechanisms and the different origins of these genes, with *Or*_*Deb2*_ directly transferred from other wild sunflower species (*H. debilis* subsp. *tardiflorus*, Velasco et al. 2012) and *Or*_*SII*_ identified within a proprietary sunflower germplasm collection (Hassan 2003), indicated that *Or*_*Anom1*_, *Or*_*Deb2*_, and *Or*_*SII*_ might represent different allelic configurations or alternatively are tightly linked non-allelic genes. The new ANOM1 resistant source would contribute to broaden the genetic basis of broomrape resistance in the cultivated sunflower pool, which is crucial due to the parasite ability to evolve physiologically, and to develop more durable and sustainable breeding strategies based on genetic resistance.

### Supplementary Information

Below is the link to the electronic supplementary material.

Fig S1. Dot plot outputs comparing the Iasnip-40 to Iasnip-105 genomic region between the *H. annuus* genome assembly HanXRQr2.0-SUNRISE and that of *H. anomalus* HanomANO2822-UBC (A), and each region against itself (B and C) (PPTX 621 kb)

Fig S2. InterPro and ProSite domain constitution of putative protein kinase genes in the Iasnip-40 to Iasnip-105 *Or*_*Anom1*_ region from the chromosome assembled genome sequence of *H. anomalus* (HanomANO2822-UBC). TAIR BlastP analyses are also shown (PPTX 13331 kb)

Fig. S3. Predominant stages of *O. cumana* development in rhizotron experiments. Broomrapes attached to ANOM1 remained in stage T1 at 14, 21, 28, and 35 dpi (A-D, respectively) and became necrotic over time (C-D). Broomrapes attached to B117 susceptible line were in stage T2 at 14 dpi (E), T3 at 21 dpi (F), and T4 at 28 dpi (G) (PPTX 2469 kb)

Fig. S4. *O. cumana* connected to the sunflower root of ANOM1 resistant line at 21 (A-C), 28 (D-F) and 35 (G-I) dpi. Transversal and longitudinal thin sections stained with TBO show xylem in light blue and phenolic compounds accumulation in green. Whole cleared samples show xylem in dark grey and phenolic compounds in brownish staining. Black scale = 250µm; white scale= 100µm (PPTX 2727 kb)

Table S1. Context sequence of SNP markers Iasnip-17 to Iasnip-47, previously developed for *Or*_*Deb2*_ fine mapping, and Iasnip-95 to Iasnip-106, developed in this study from ANOM1 and P21 cloned fragments (Excel sheet 1), and PCR primer sequences used to amplify the ANOM1 and P21 DNA fragments (Excel sheet 2) (XLSX 15 kb)

Table S2. Mapping population SNP genotyping data (A= Allele from the resistant parental line; B= Allele from the susceptible parental; H= Heterozygous; D= A or H; C= B or H) and score of the OrANOM1 gene inferred from phenotypic data (A=Homozygous resistant; B=Homozygous susceptible; H=Heterozygous; D= A or H)(XLSX 37 kb)

Table S3. Linkage map details and location of the OrAnom1 gene (XLSX 15 kb)

Table S4. Results of tBlastn of the annotated proteins from H. annuus-HanXRQr2.0-SUNRISE from the Chr 4 Iasnip-40 to Iasnip-105 region against the Iasnip-40 to Iasnip-105 genome sequence in the H. anomalus HanomANO2822-UBC assembly. Hits showing the highest identity are highlighted (XLSX 49 kb)

Table S5. Putative protein coding genes (Excel sheet 1) and their translated amino acid sequences (Excel sheet 2) in the Iasnip-40 to Iasnip-105 region from the chromosome assembled genome sequence of H. anomalus (HanomANO2822-UBC) (XLSX 29 kb)

## Data Availability

PCR primer sequences, SNP context sequences, tBlastn results, aminoacid sequences of the putative protein coding genes in the target region, and the SNP data set from the mapping population are available as supplementary material. The *H. anomalus* chromosome-level genome sequence has been sent to NCBI under BioProject PRJNA1000454 and finally deposited at DDBJ/ENA/GenBank under the accession JAWUZI000000000. The version used in this manuscript is JAWUZI010000000.

## Abbreviations

cM: CentiMorgans
Chr: Chromosome
LRR: Leucine-rich repeat
RING: Really interesting new gene
RLK: Receptor-like kinase
RLP: Receptor-like protein
SWEET: Sugars will eventually be exported transporters
UPS: Ubiquitin–proteasome system
