# Supplementary material for: Development and characterization of a new sunflower source of resistance to race G of Orobanche cumana Wallr. derived from Helianthus anomalus
Source: Theor Appl Genet. 2024 Feb 22;137(3):56. doi: 10.1007/s00122-024-04558-4 (PMC10884359; doi:10.1007/s00122-024-04558-4)
Supplement: Supplementary file 1 — Fig S1. Dot plot outputs comparing the Iasnip-40 to Iasnip-105 genomic region between the H. annuus genome assembly HanXRQr2.0-SUNRISE and that of H. anomalus HanomANO2822-UBC (A), and each region against itself (B and C) (PPTX 621 kb) [file 122_2024_4558_MOESM1_ESM.pptx]

## Slide 1
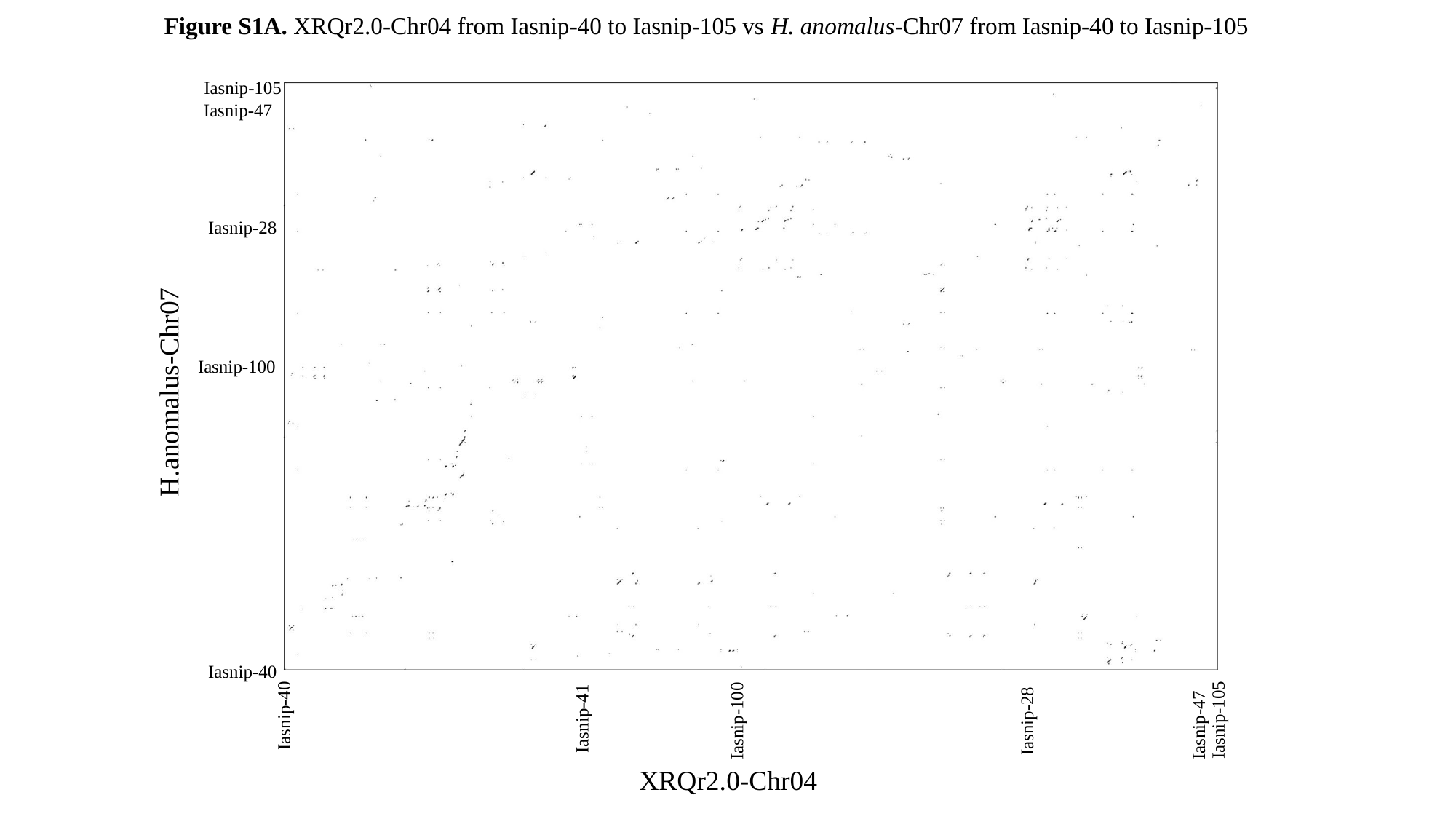

Figure S1A. XRQr2.0-Chr04 from Iasnip-40 to Iasnip-105 vs H. anomalus-Chr07 from Iasnip-40 to Iasnip-105
Iasnip-105
H.anomalus-Chr07
Iasnip-40
Iasnip-40
Iasnip-105
XRQr2.0-Chr04
Iasnip-47
Iasnip-28
Iasnip-100
Iasnip-28
Iasnip-41
Iasnip-47
Iasnip-100

## Slide 2
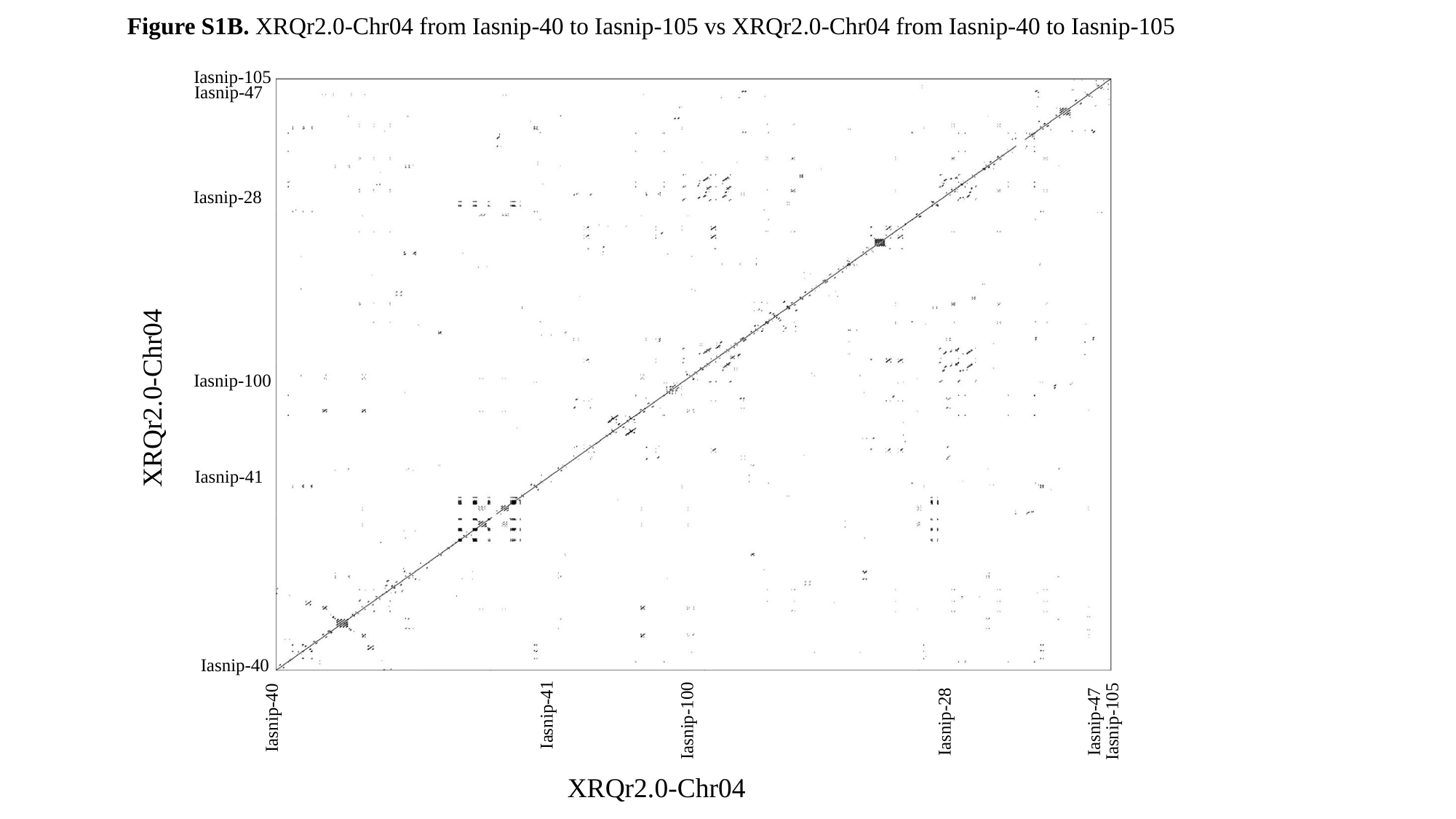

# Figure S1B. XRQr2.0-Chr04 from Iasnip-40 to Iasnip-105 vs XRQr2.0-Chr04 from Iasnip-40 to Iasnip-105
Iasnip-105
XRQr2.0-Chr04
Iasnip-40
Iasnip-40
Iasnip-105
Iasnip-100
Iasnip-41
Iasnip-41
Iasnip-100
Iasnip-47
Iasnip-28
Iasnip-47
Iasnip-28
XRQr2.0-Chr04

## Slide 3
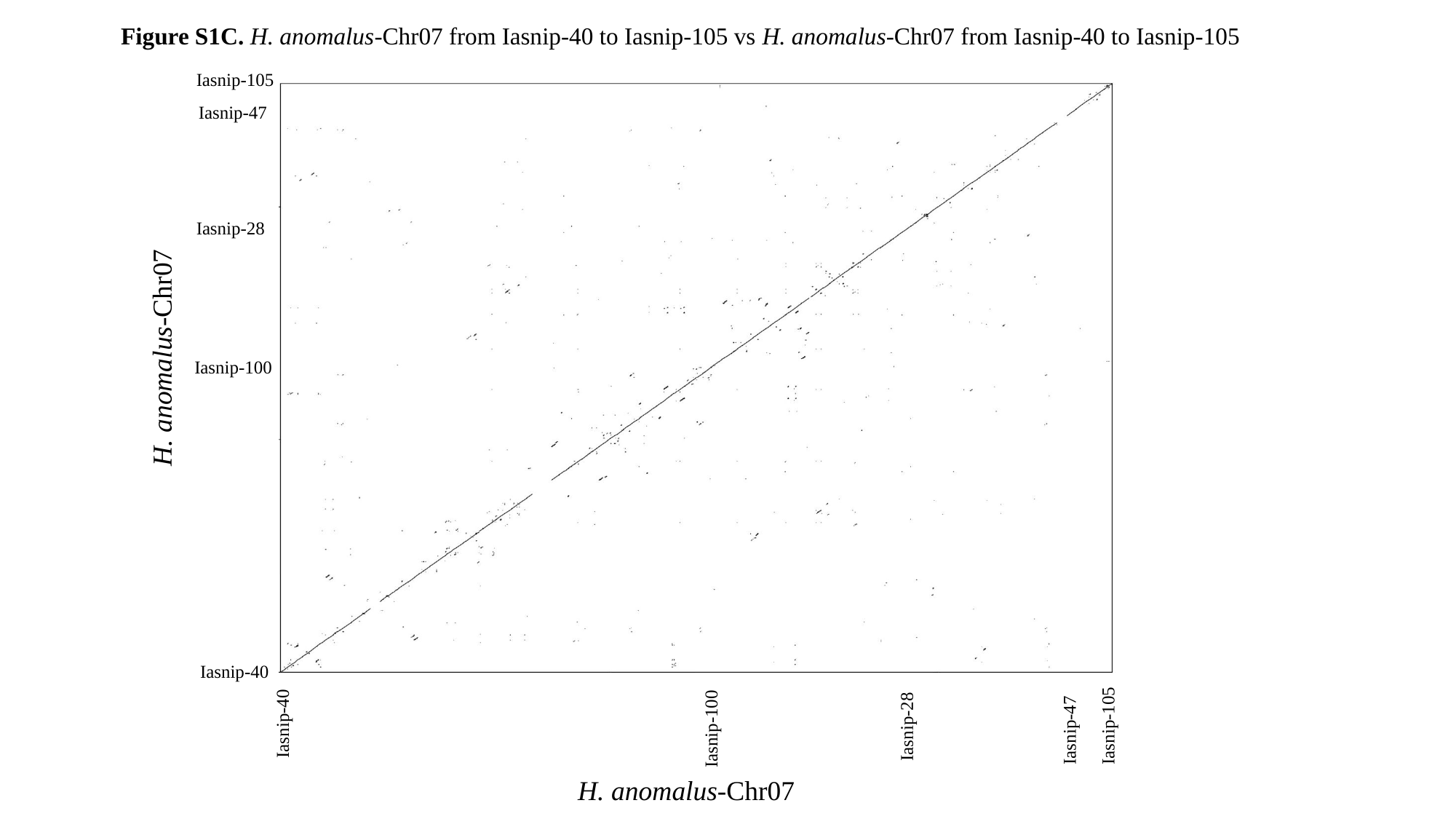

Figure S1C. H. anomalus-Chr07 from Iasnip-40 to Iasnip-105 vs H. anomalus-Chr07 from Iasnip-40 to Iasnip-105
Iasnip-105
H. anomalus-Chr07
Iasnip-40
Iasnip-40
Iasnip-105
H. anomalus-Chr07
Iasnip-100
Iasnip-100
Iasnip-47
Iasnip-28
Iasnip-28
Iasnip-47
