# Supplementary material for: Development and characterization of a new sunflower source of resistance to race G of Orobanche cumana Wallr. derived from Helianthus anomalus
Source: Theor Appl Genet. 2024 Feb 22;137(3):56. doi: 10.1007/s00122-024-04558-4 (PMC10884359; doi:10.1007/s00122-024-04558-4)
Supplement: Supplementary file 2 — Fig S2. InterPro and ProSite domain constitution of putative protein kinase genes in the Iasnip-40 to Iasnip-105 OrAnom1 region from the chromosome assembled genome sequence of H. anomalus (HanomANO2822-UBC). TAIR BlastP analyses are also shown (PPTX 13331 kb) [file 122_2024_4558_MOESM2_ESM.pptx]

## Slide 1
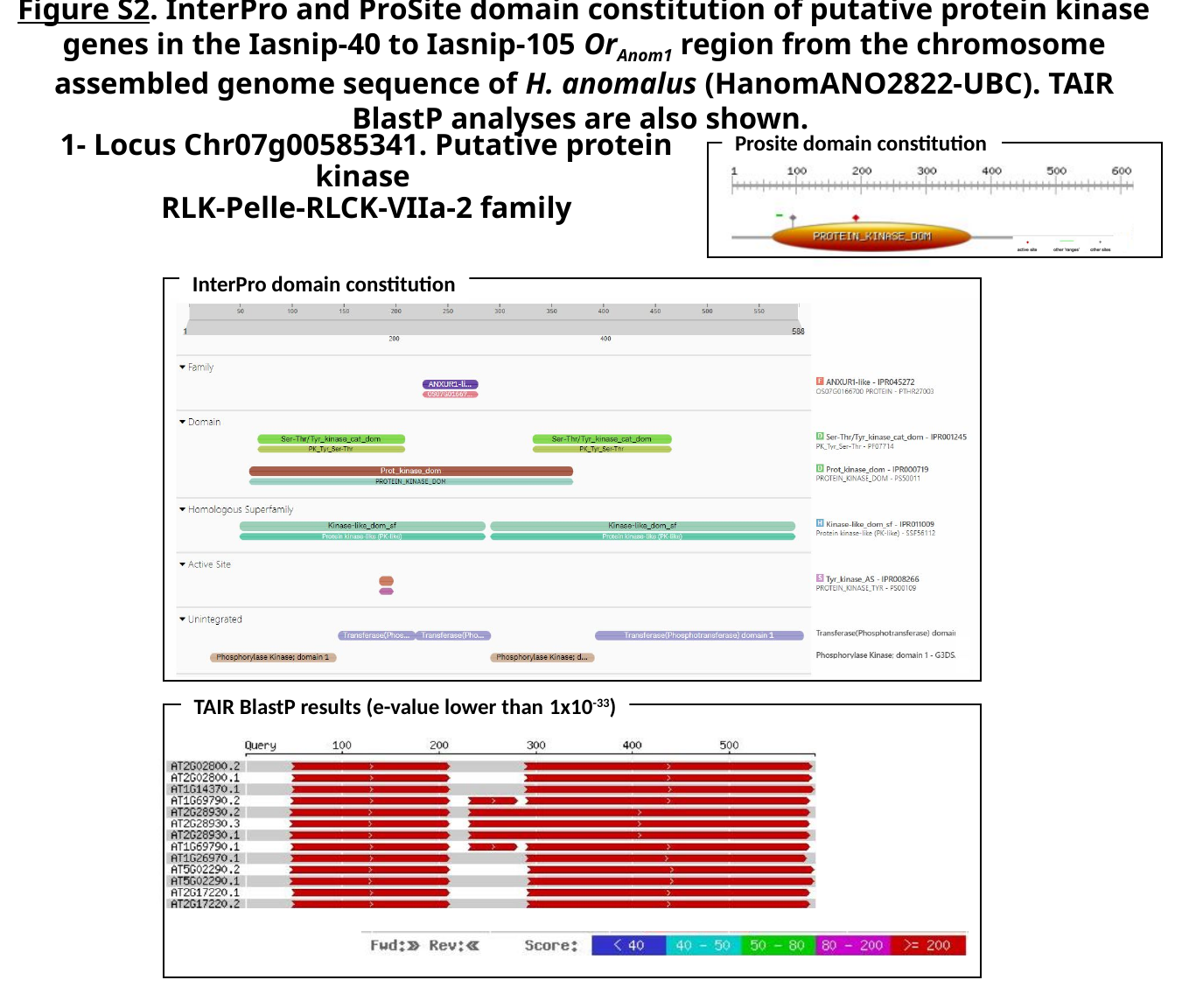

Figure S2. InterPro and ProSite domain constitution of putative protein kinase genes in the Iasnip-40 to Iasnip-105 OrAnom1 region from the chromosome assembled genome sequence of H. anomalus (HanomANO2822-UBC). TAIR BlastP analyses are also shown.
Prosite domain constitution
1- Locus Chr07g00585341. Putative protein kinase
RLK-Pelle-RLCK-VIIa-2 family
InterPro domain constitution
TAIR BlastP results (e-value lower than 1x10^-33^)

## Slide 2
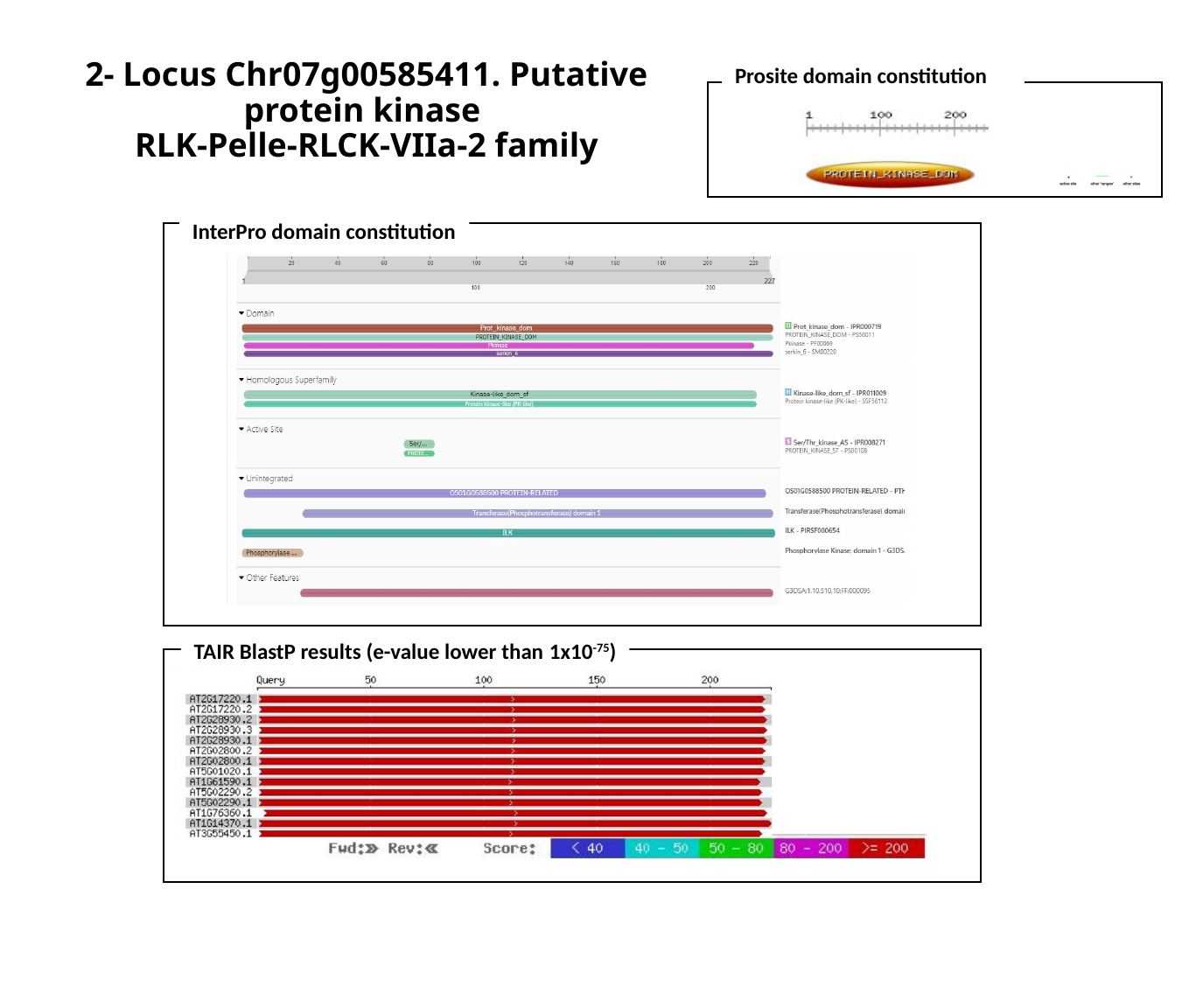

Prosite domain constitution
2- Locus Chr07g00585411. Putative protein kinase
RLK-Pelle-RLCK-VIIa-2 family
InterPro domain constitution
TAIR BlastP results (e-value lower than 1x10^-75^)

## Slide 3
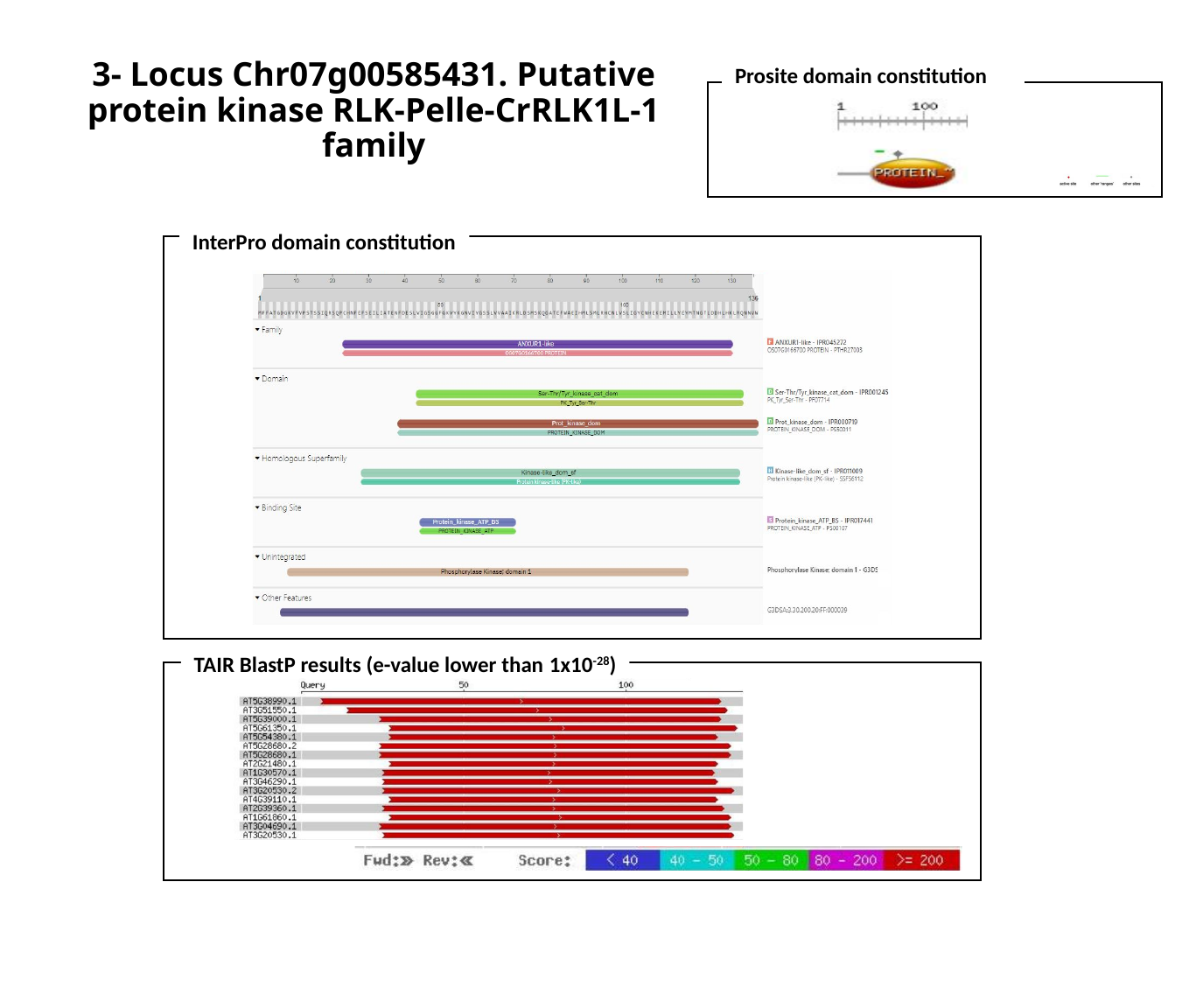

Prosite domain constitution
3- Locus Chr07g00585431. Putative protein kinase RLK-Pelle-CrRLK1L-1 family
InterPro domain constitution
TAIR BlastP results (e-value lower than 1x10^-28^)

## Slide 4
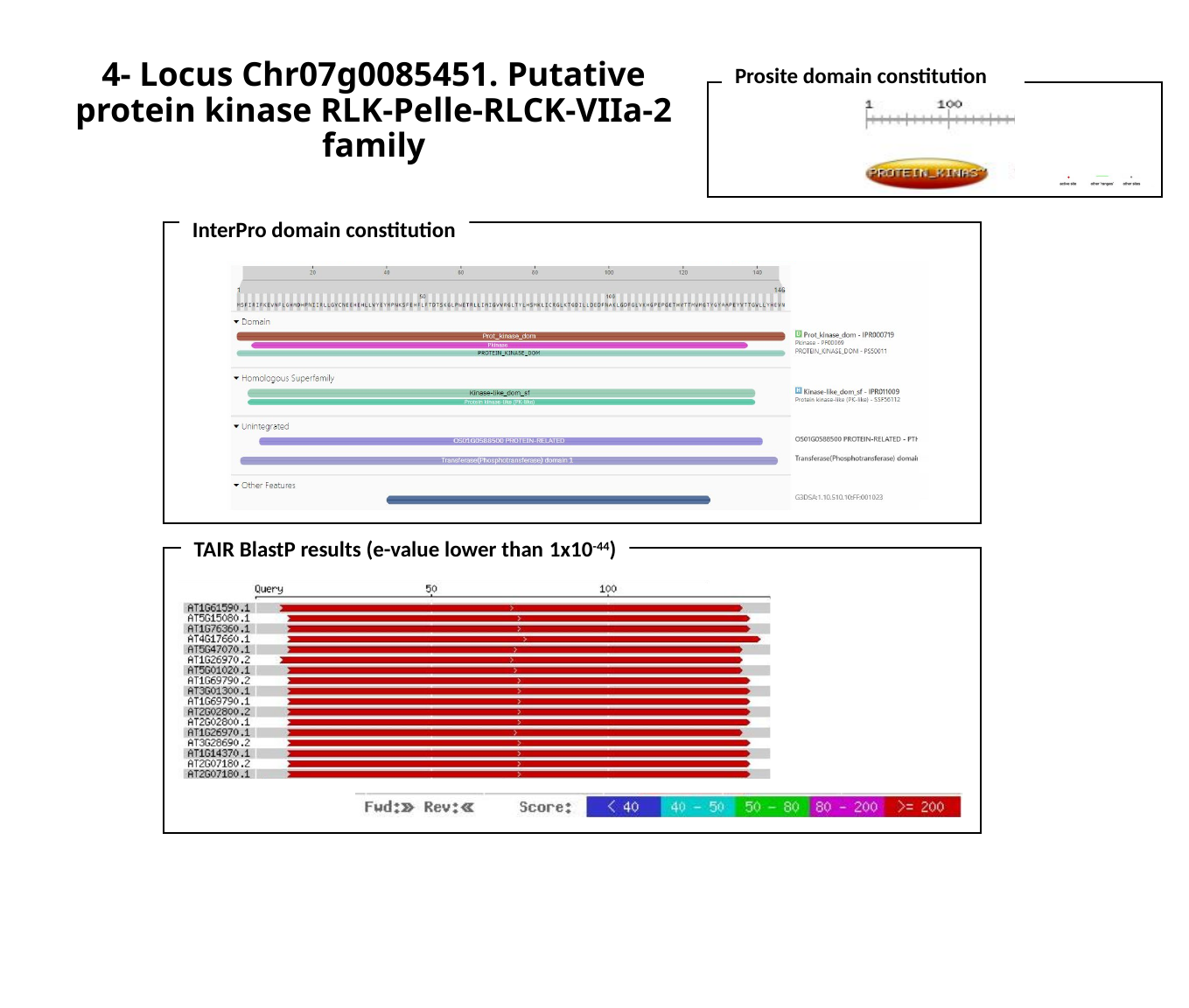

Prosite domain constitution
4- Locus Chr07g0085451. Putative protein kinase RLK-Pelle-RLCK-VIIa-2 family
InterPro domain constitution
TAIR BlastP results (e-value lower than 1x10^-44^)

## Slide 5
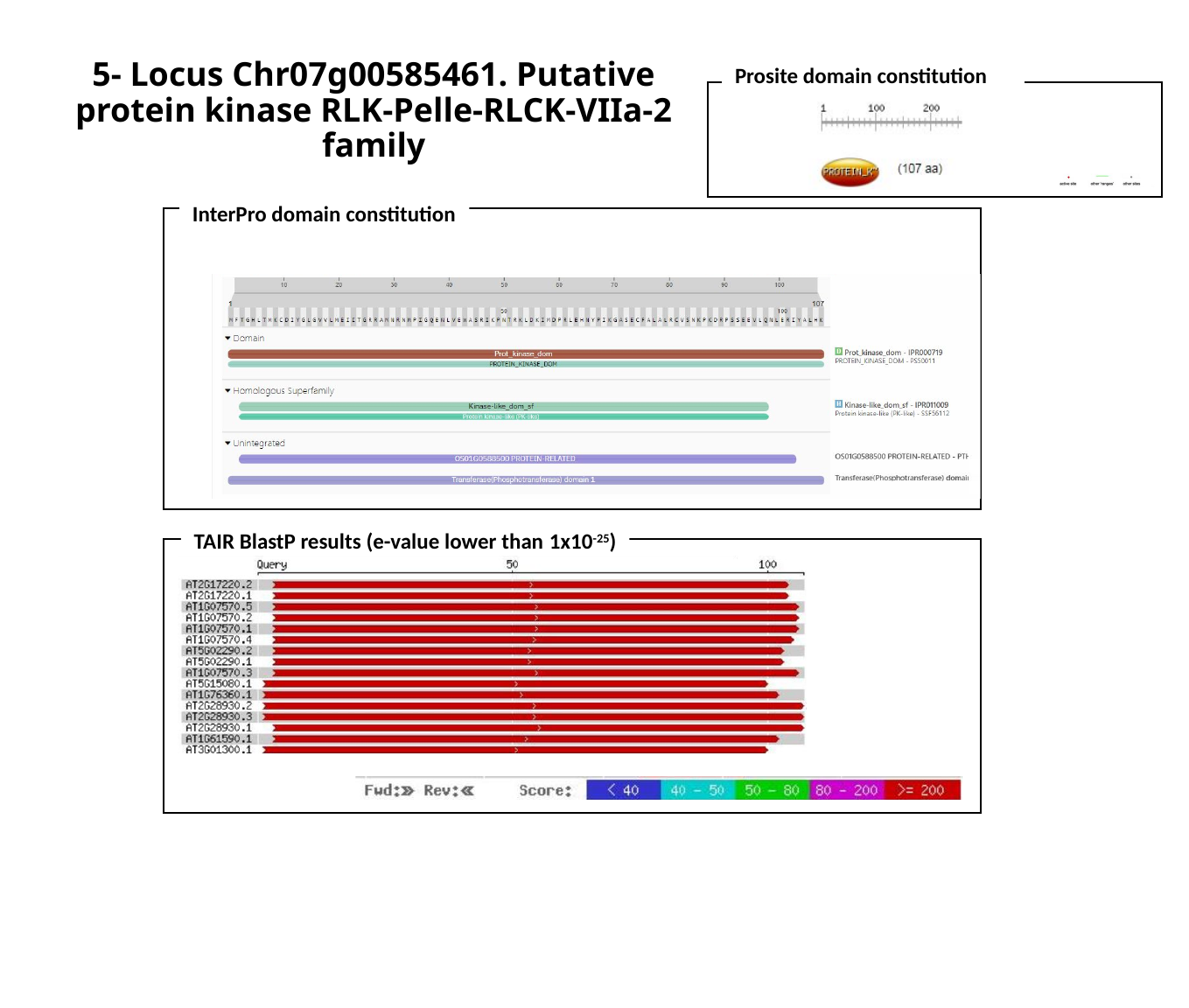

Prosite domain constitution
5- Locus Chr07g00585461. Putative protein kinase RLK-Pelle-RLCK-VIIa-2 family
InterPro domain constitution
TAIR BlastP results (e-value lower than 1x10^-25^)

## Slide 6
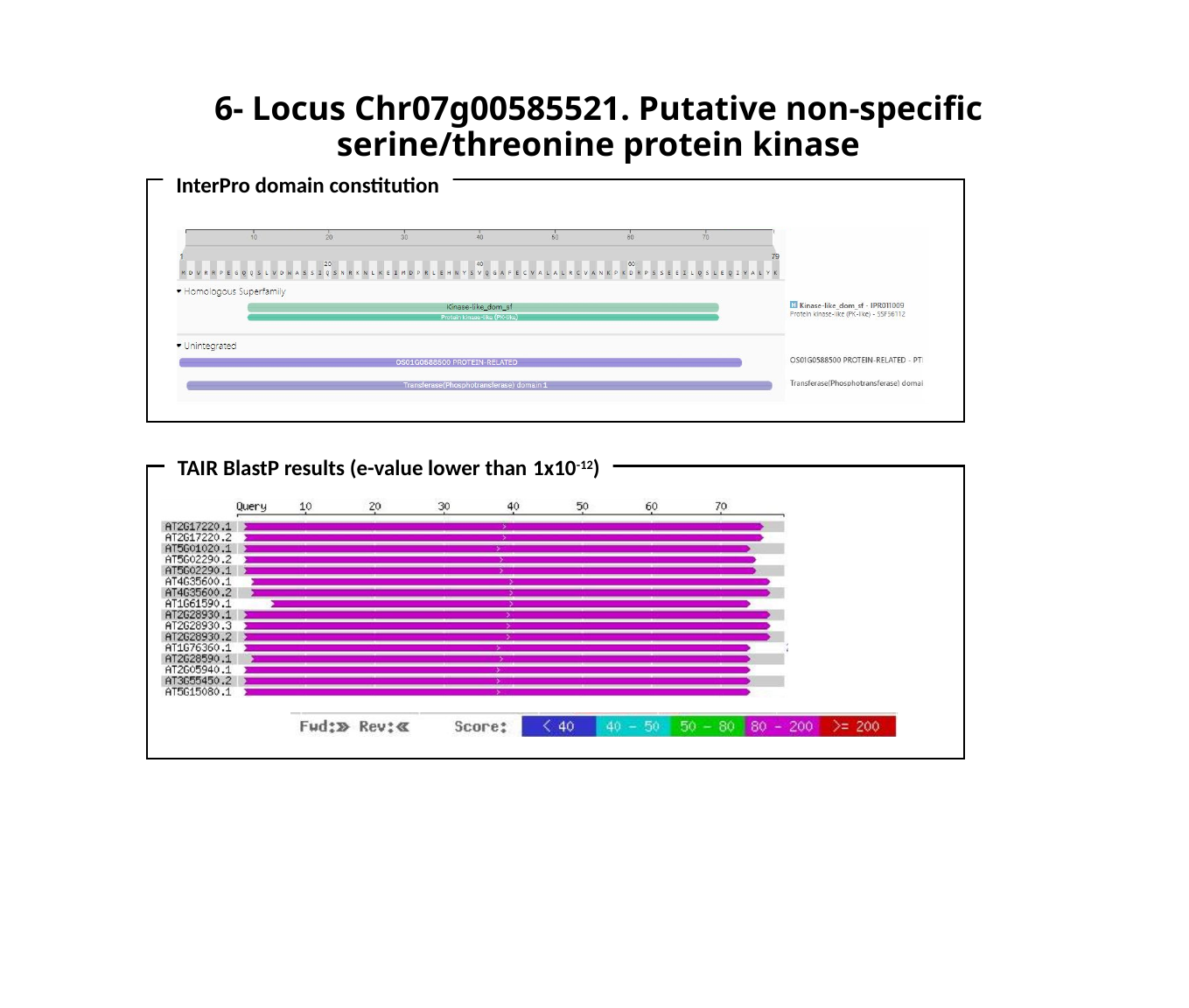

6- Locus Chr07g00585521. Putative non-specific serine/threonine protein kinase
InterPro domain constitution
TAIR BlastP results (e-value lower than 1x10^-12^)

## Slide 7
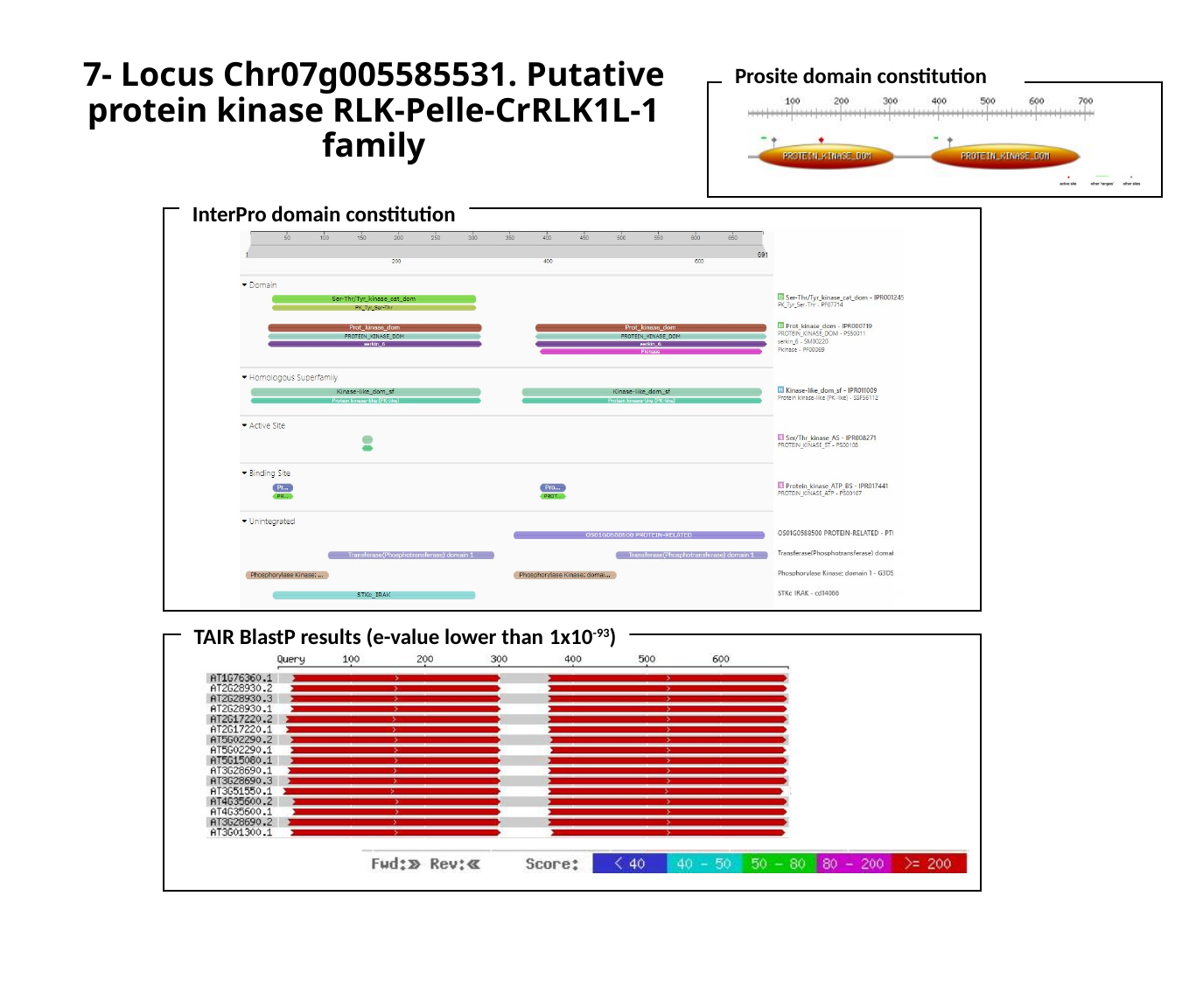

Prosite domain constitution
7- Locus Chr07g005585531. Putative protein kinase RLK-Pelle-CrRLK1L-1 family
InterPro domain constitution
TAIR BlastP results (e-value lower than 1x10^-93^)

## Slide 8
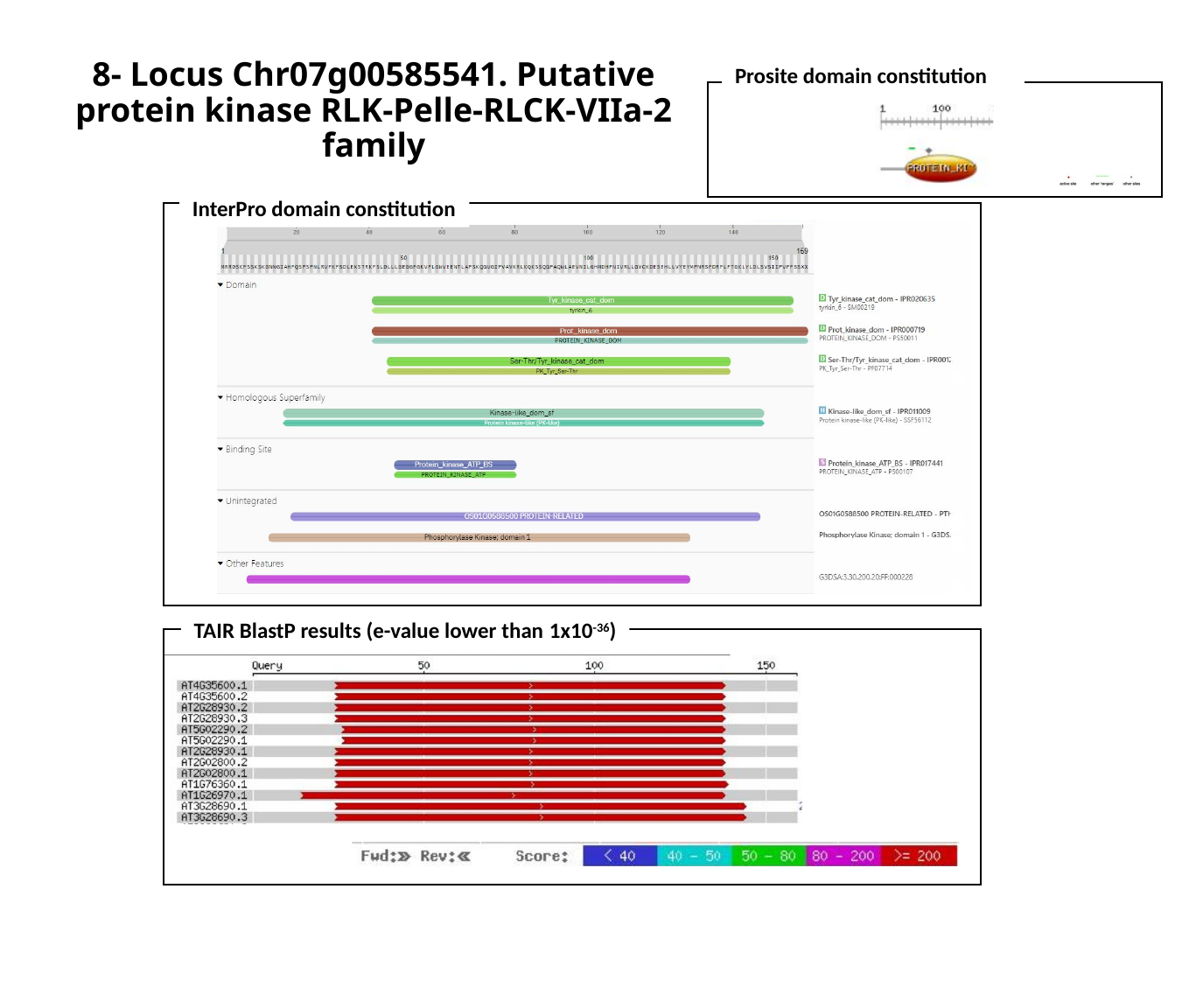

Prosite domain constitution
8- Locus Chr07g00585541. Putative protein kinase RLK-Pelle-RLCK-VIIa-2 family
InterPro domain constitution
TAIR BlastP results (e-value lower than 1x10^-36^)

## Slide 9
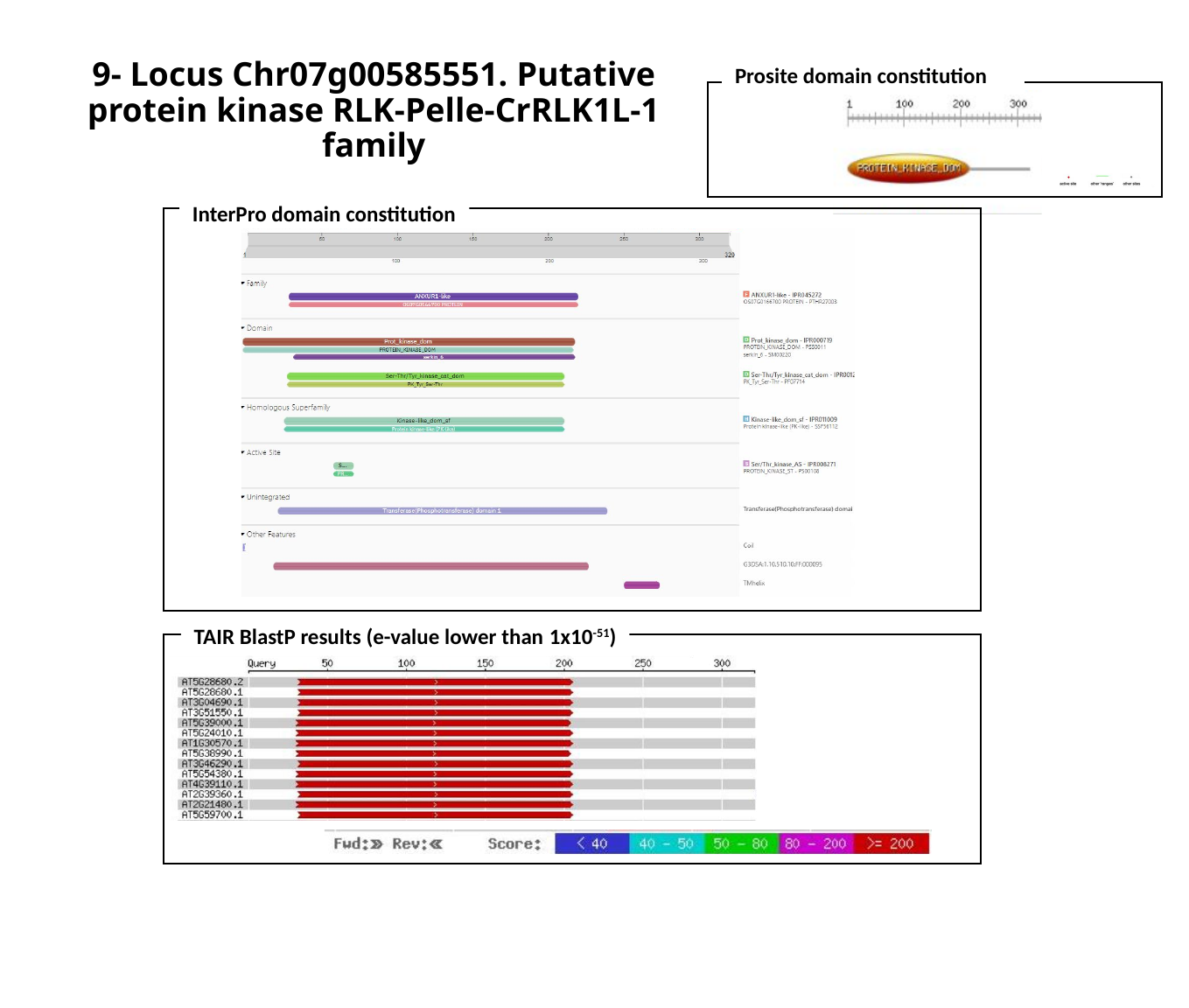

Prosite domain constitution
9- Locus Chr07g00585551. Putative protein kinase RLK-Pelle-CrRLK1L-1 family
InterPro domain constitution
TAIR BlastP results (e-value lower than 1x10^-51^)

## Slide 10
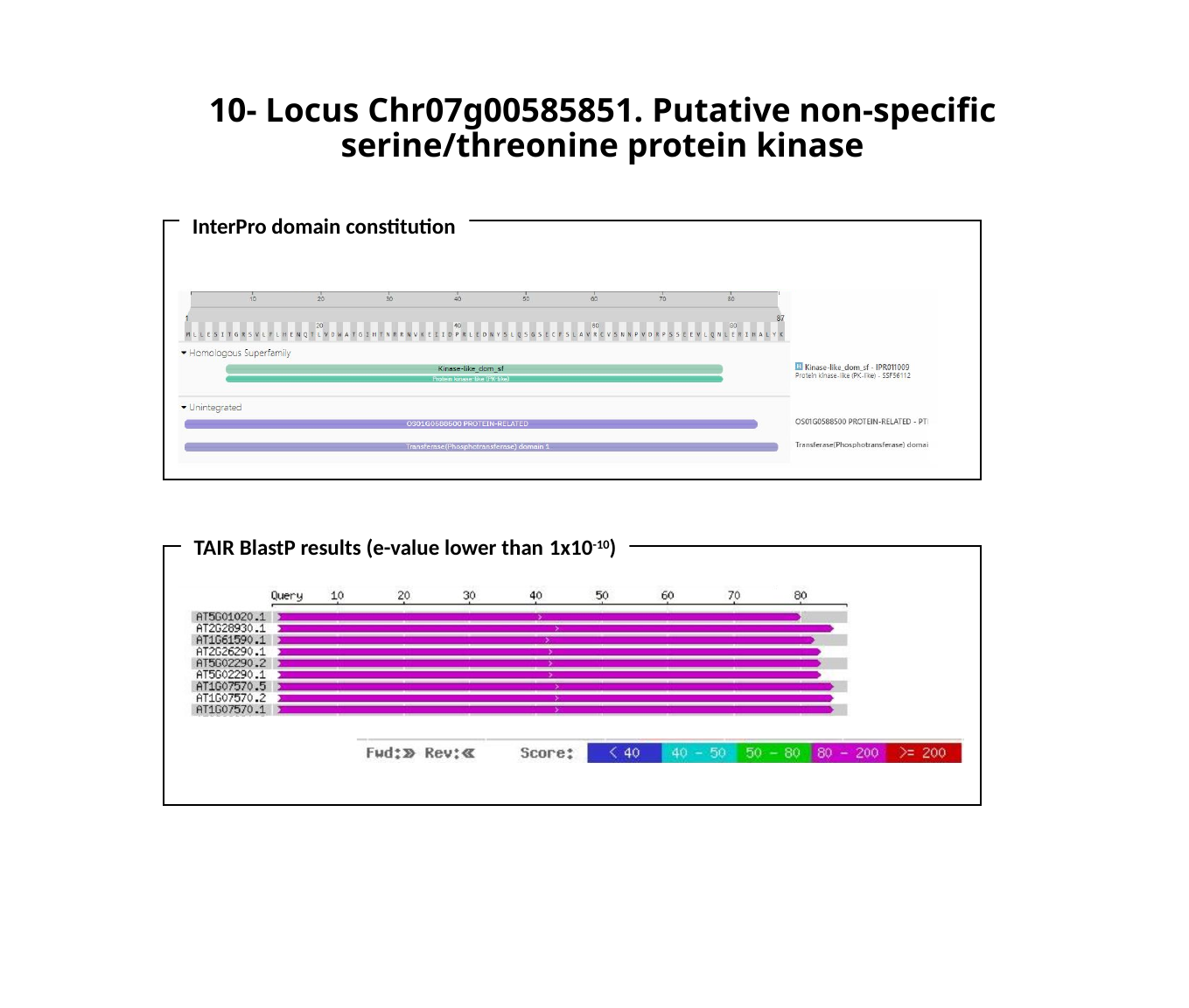

10- Locus Chr07g00585851. Putative non-specific serine/threonine protein kinase
InterPro domain constitution
TAIR BlastP results (e-value lower than 1x10^-10^)

## Slide 11
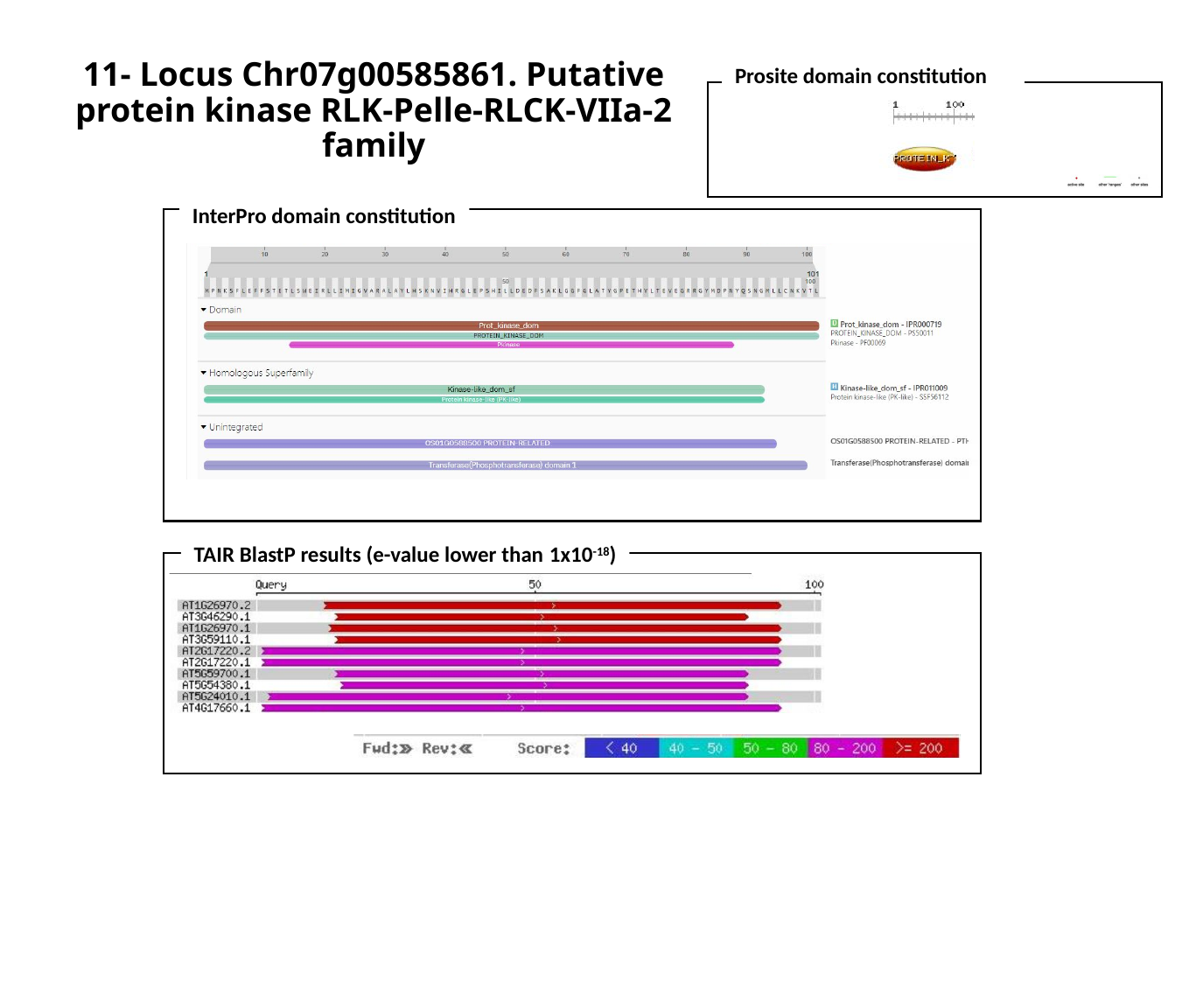

Prosite domain constitution
11- Locus Chr07g00585861. Putative protein kinase RLK-Pelle-RLCK-VIIa-2 family
InterPro domain constitution
TAIR BlastP results (e-value lower than 1x10^-18^)
