# Supplementary material for: Development and characterization of a new sunflower source of resistance to race G of Orobanche cumana Wallr. derived from Helianthus anomalus
Source: Theor Appl Genet. 2024 Feb 22;137(3):56. doi: 10.1007/s00122-024-04558-4 (PMC10884359; doi:10.1007/s00122-024-04558-4)
Supplement: Supplementary file 3 — Fig. S3. Predominant stages of O. cumana development in rhizotron experiments. Broomrapes attached to ANOM1 remained in stage T1 at 14, 21, 28, and 35 dpi (A-D, respectively) and became necrotic over time (C-D). Broomrapes attached to B117 susceptible line were in stage T2 at 14 dpi (E), T3 at 21 dpi (F), and T4 at 28 dpi (G) (PPTX 2469 kb) [file 122_2024_4558_MOESM3_ESM.pptx]

## Slide 1
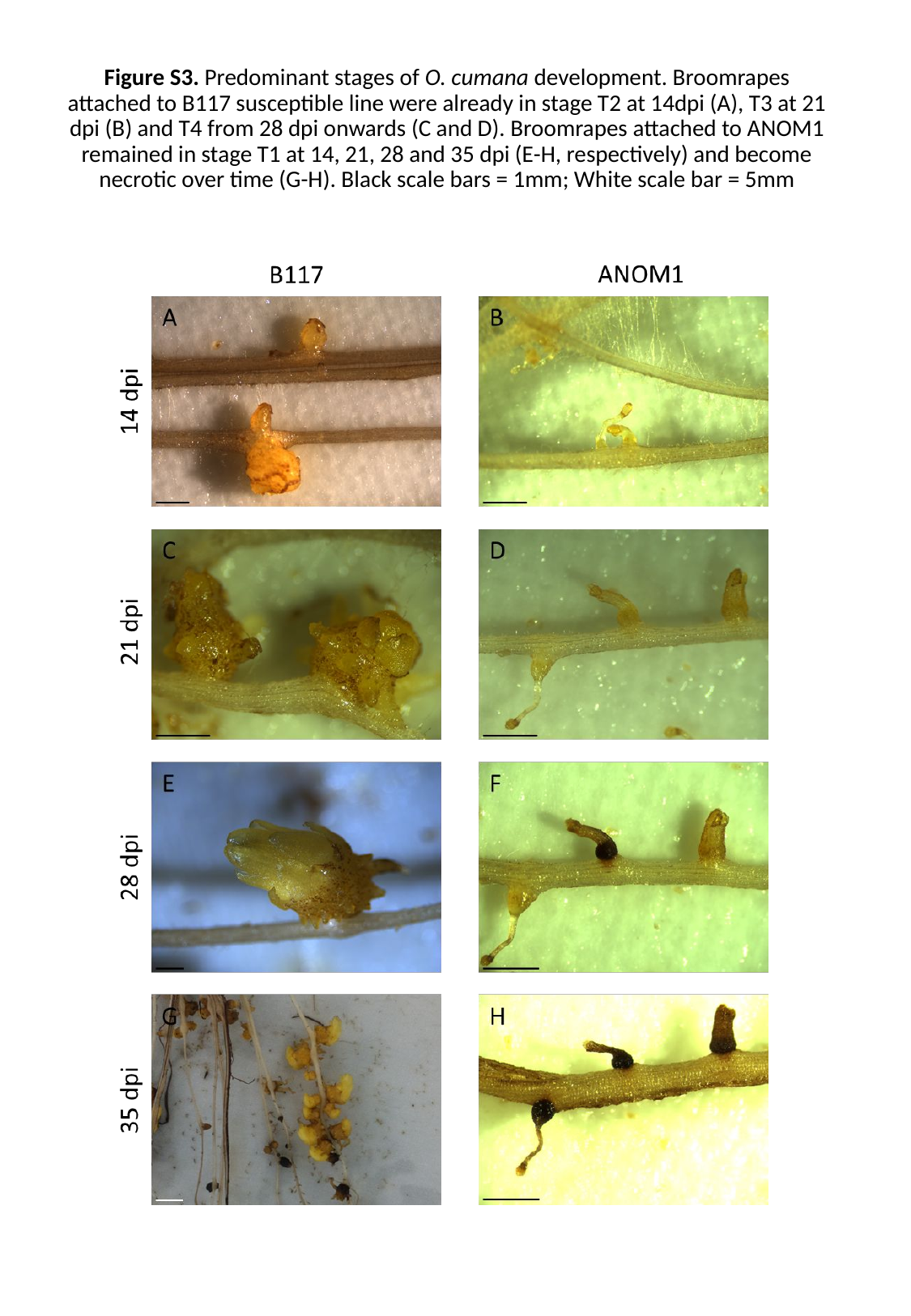

Figure S3. Predominant stages of O. cumana development. Broomrapes attached to B117 susceptible line were already in stage T2 at 14dpi (A), T3 at 21 dpi (B) and T4 from 28 dpi onwards (C and D). Broomrapes attached to ANOM1 remained in stage T1 at 14, 21, 28 and 35 dpi (E-H, respectively) and become necrotic over time (G-H). Black scale bars = 1mm; White scale bar = 5mm
