# Supplementary material for: Development and characterization of a new sunflower source of resistance to race G of Orobanche cumana Wallr. derived from Helianthus anomalus
Source: Theor Appl Genet. 2024 Feb 22;137(3):56. doi: 10.1007/s00122-024-04558-4 (PMC10884359; doi:10.1007/s00122-024-04558-4)
Supplement: Supplementary file 4 — Fig. S4. O. cumana connected to the sunflower root of ANOM1 resistant line at 21 (A-C), 28 (D-F) and 35 (G-I) dpi. Transversal and longitudinal thin sections stained with TBO show xylem in light blue and phenolic compounds accumulation in green. Whole cleared samples show xylem in dark grey and phenolic compounds in brownish staining. Black scale = 250µm; white scale= 100µm (PPTX 2727 kb) [file 122_2024_4558_MOESM4_ESM.pptx]

## Slide 1
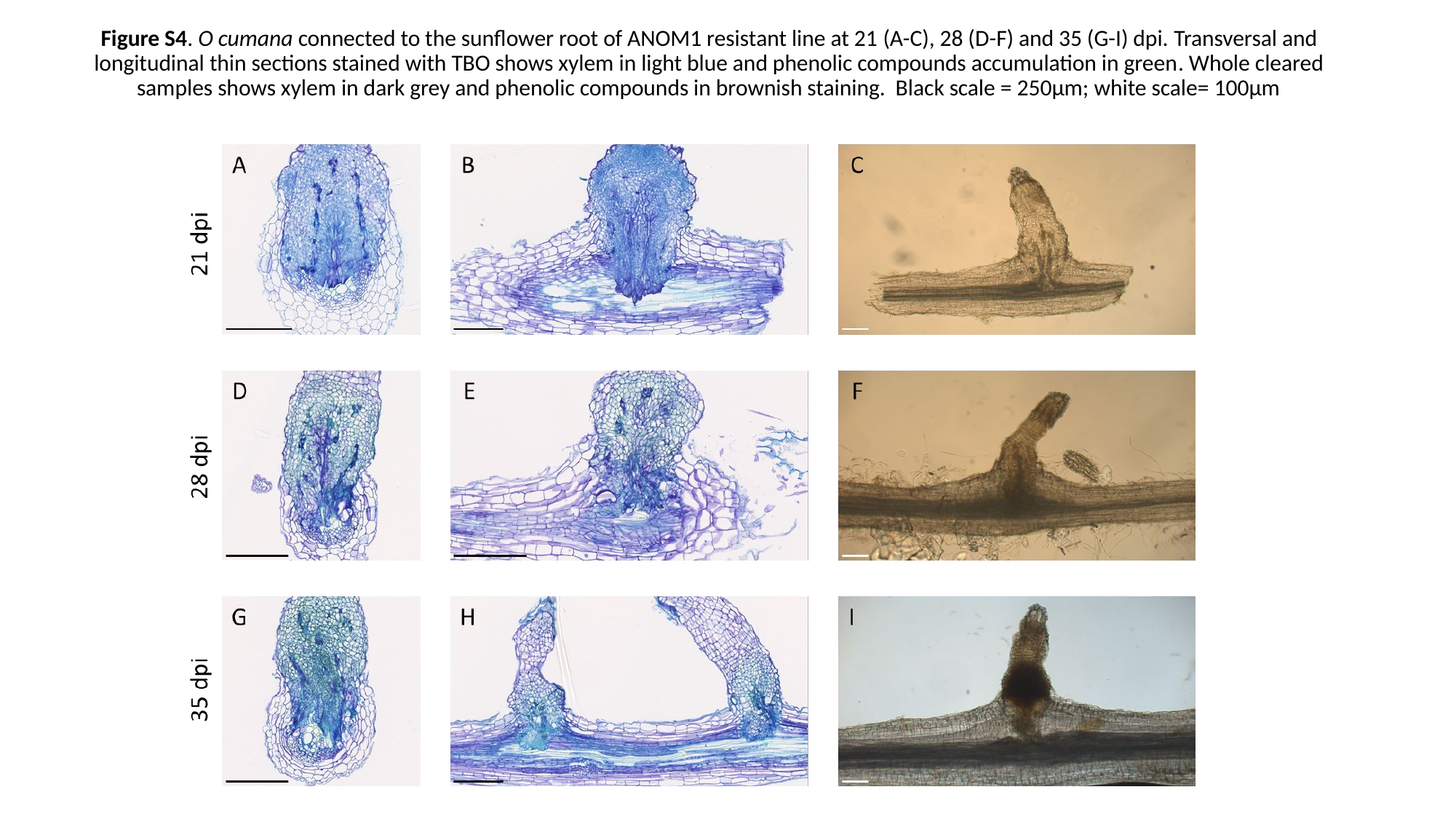

Figure S4. O cumana connected to the sunflower root of ANOM1 resistant line at 21 (A-C), 28 (D-F) and 35 (G-I) dpi. Transversal and longitudinal thin sections stained with TBO shows xylem in light blue and phenolic compounds accumulation in green. Whole cleared samples shows xylem in dark grey and phenolic compounds in brownish staining. Black scale = 250µm; white scale= 100µm
